# Personalized behavior management as a replacement for medications for pain control and mood regulation

**DOI:** 10.1038/s41598-021-99803-x

**Published:** 2021-10-13

**Authors:** Dmitry M. Davydov, Carmen M. Galvez-Sánchez, Casandra Isabel Montoro, Cristina Muñoz Ladrón de Guevara, Gustavo A. Reyes del Paso

**Affiliations:** 1 Laboratory of Neuroimmunopathology, Institute of General Pathology and Pathophysiology, Russian Academy of Sciences, 8 Baltiyskaia ul., Moscow, 125315 Russia; 2 University of Jaén Hospital, FIBAO, Jaén, Spain; 3 Department of Psychology, University of Jaén, Jaén, Spain

**Keywords:** Emotion, Stress and resilience, Human behaviour, Fibromyalgia

## Abstract

A lack of personalized approaches in non-medication pain management has prevented these alternative forms of treatment from achieving the desired efficacy. One hundred and ten female patients with fibromyalgia syndrome (FMS) and 60 healthy women without chronic pain were assessed for severity of chronic or retrospective occasional pain, respectively, along with alexithymia, depression, anxiety, coping strategies, and personality traits. All analyses were conducted following a ‘resource matching’ hypothesis predicting that to be effective, a behavioral coping mechanism diverting or producing cognitive resources should correspond to particular mechanisms regulating pain severity in the patient. Moderated mediation analysis found that extraverts could effectively cope with chronic pain and avoid the use of medications for pain and mood management by lowering depressive symptoms through the use of distraction mechanism as a habitual (‘out-of-touch-with-reality’) behavior. However, introverts could effectively cope with chronic pain and avoid the use of medications by lowering catastrophizing through the use of distraction mechanism as a situational (‘in-touch-with-reality’) behavior. Thus, personalized behavior management techniques applied according to a mechanism of capturing or diverting the main individual ‘resource’ of the pain experience from its ‘feeding’ to supporting another activity may increase efficacy in the reduction of pain severity along with decreasing the need for pain relief and mood-stabilizing medications.

## Introduction

High rates of overdoses on painkillers in patients with chronic pain, as seen in the opioid epidemics in the USA, begs for new approaches in effective and harmless pain control so as to reduce reliance on medications like analgesics, antidepressants, and anxiolytics^1^. However, non-drug approaches in pain management, such as physical exercise (e.g., walking and yoga) and psychological methods (e.g., cognitive distraction) have suffered from variability in outcome efficacy and, as alternative behavioral forms of treatment (i.e., as the replacement of medication), may only be effective in patients who are specifically primed to such interventions (a main *‘resource matching’ hypothesis*)^2^.

For example, patients suffering mainly from the mental component of chronic pain associated with low or high vagus (re)activity were found to benefit from, respectively, walking due to its positive effects on the Vagus nerve (i.e., through the increase of scarce resources that are required for neutralizing pain) or from yoga claiming parasympathetic resources and thus diverting available but restricted resources feeding the specific component of pain to another “consumer”, such as another mental activity^2,3^. In concordance with these findings, it was previously hypothesized that behavioral methods in pain management can also be effective if they appeal to pain-regulation mechanisms consistent with pain causality mechanisms, e.g., to maladaptive affect-regulation as in chronic pain associated mainly with its affective (emotional or depressive) experience or to maladaptive thinking-regulation as in chronic pain associated mainly with its psychotic-like or catastrophizing experience^4,5^. Specifically, a pain management approach can appeal to available but restricted resources to decrease severity of the respective pain component depending on (i.e., fed by) these resources, such as pain-related affect or thoughts (i.e., *a mechanism “diverting resources”* from the support of pain severity), or can increase lacked resources, for which a deficit increases respective pain components, such as pain-related affect or thoughts (i.e., *a mechanism “producing resources”* for decreasing severity of pain). If the hypotheses are confirmed, these mechanisms can be applied to individualize different physiological and psychological treatments for pain management.

Thus, according to the main ‘resource matching’ hypothesis, to be effective in outcomes including medication replacement, a behavioral pain-coping mechanism diverting or producing resources should correspond to particular mechanisms regulating pain severity in the patient. For example, it was suggested that cognitive distraction as a coping strategy in the acute pain condition is effective mainly in individuals with high levels of pain catastrophizing, i.e., in those with disorganized cognitive activity, when pain-directed attention utilizes cognitive resources for ‘feeding’ pain intensity^6,7^. In patients with chronic pain of different etiology, pain catastrophizing was found to be associated with maladaptive (i.e., disorganized, bizarre, or disoriented) thinking as in a psychotic-like syndrome, i.e., in a condition characterized by a high deficit in the core motivational and ego-mastery mechanisms, which can result in medication overuse with substance addiction problems^4,5^. Thus, the process of pain catastrophizing seems to be managed by an avoidance motivation mechanism for coping with pain that is associated with general withdrawal strategies or low ‘energy’ for approach behaviors. It may be suggested that distraction strategies lower the experience of pain by suppressing a tendency to catastrophize as a cognitive mechanism associated with pain aggravation more so than being a direct effect on the somatic sensation of pain^8^. So, if a distraction-based strategy decreases catastrophizing, this mechanism could be an important factor in decreasing a patient's pain experience. This mechanism predicted by the ‘resource matching’ hypothesis deserves additional investigation given perceived pain (assessed by, e.g., the McGill Pain Questionnaire) was found to be positively correlated with catastrophizing^9–11^. Thus, according to the ‘resource matching’ hypothesis, people may benefit (in the form of decrease of pain severity and medication use) from a distraction-based coping style if a pain-amplifying mechanism is related to greater attention to pain, i.e., pain catastrophizing^4,12^.

Besides catastrophizing, depression is also considered an aggravating factor associated with the severity of chronic pain syndromes^4,11,13^. Specifically, this pain component is associated with maladaptive affect-regulation including deep concerns about physical symptoms, somatization and conversion reactions, intentional and unintentional help-seeking strategies (social approach motivation), excessive ‘energy’ for other approach behaviors, which can result in medication overuse, but without other substance addiction problems^4^. Cognitive resources are burdened in depression by persistent ruminations or excessive and intrusive thoughts about negative experiences and feelings, which are the key ‘adaptive’ process underlying this affective condition, especially in those with chronic pain^14–16^. Rumination mediates the path of disrupted attentional control to depression severity thus maintaining depressive symptoms and pointing to this mechanism as a potential target of intervention for protecting against pain severity in cases where this affective component leads to the aggravation of pain^17,18^. This pain aggravation pathway may be effectively altered by attention distraction strategies in the form of habitual behavior, represented by the externally oriented thinking (EOT) facet of alexithymia, against affective stimuli, including the affective component of pain, (i.e., a reflexive or conditioned self-management of affect), because only this facet of alexithymia was found to protect mood in the face of negative events in contrast to other alexithymia components (i.e., difficulty identifying and describing feelings)^19–22^.

This cognitive or attentional component of alexithymia represents a tendency to focus attention on external events and activities with high inhibitory control for their emotive component or associated internal emotional experiences, as a habitual cognitive style monitoring environment as aversive and correlating negatively with depressive concrete-experiential rumination^19,23,24^. It can be the background for the realization of the similar pain protection mechanism as described above for the situational cognitive distraction coping mechanism. The EOT facet of alexithymia was found to determine a mood resilience mechanism through the central dissociation of actual symptoms of affective and somatic conditions, such as depression and pain (evident from lower complaints on respective scales in those persons with high EOT), from socially desirable reports/cognitive evaluations of these conditions (evident from higher complaints on respective scales in those persons with high EOT) as their impression management style of responding^19,20,25^. According to the ‘resource matching’ hypothesis, high inhibition abilities found in persons with high EOT style could be a part of the mechanism capturing resources supporting depressive thoughts related to pain in favor of cognitive processes distinct from pain and thus decreasing pain severity and medication use as outcomes^26,27^.

As well, personality traits or habitual patterns of behavior, thought, and emotion, such as neuroticism (a negative affectivity with hypervigilance toward somatic sensations and their interpretation as negative or threatening), psychoticism (lack of sociability and self-centered impulsivity), and extraversion (a habitual behavior with concentration of attention on external objects, excitement-seeking, and stress immunity), may affect the reallocation of resources from ‘feeding’ pain to both cognitive distraction mechanisms, a situational pain-related attention diverting response, and a habitual affect-related cognitive processing style, such as EOT, and thus moderate their protective effects on the pain experience and medication use^4,28–34^. For example, extraversion as a behavior trait determining habitual (e.g., compulsive) externally oriented behaviors can moderate (match or mismatch) EOT as a cognitive trait determining habitual (e.g., obsessive) externally oriented thoughts^35^. Indeed, some findings have given indirect evidence that effects of EOT on pain intensity and severity can be moderated in different populations to show either a pain risk, neutral, or protection capability^21,22,36–40^. However, moderation and mediation effects of pain catastrophizing, depression with associated rumination, and personality traits on the relationships between chronic pain severity in its psychotic-like and affective domains and the situational and habitual cognitive distraction strategies in self-management of pain intensity have not been well explored, especially according to the ‘resource matching’ hypothesis. Specifically, a moderation mechanism should be related to the competition for restricted cognitive resources between the distraction processes and the pain catastrophizing/depressive rumination processes in favor of the first (*a ‘competition’ mechanism for the ‘common resources’* as one of specific mechanisms predicted by the ‘resource matching’ hypothesis). A mediation mechanism should be related to more resources released in those with higher pain catastrophizing/depressive rumination to favor these distraction mechanisms to cope with pain (*a ‘capturing’ mechanism of the ‘common resources’* as another specific mechanisms predicted by the ‘resource matching’ hypothesis). This study was conducted to verify these ‘competition’ and ‘capturing’ mechanisms related to the ‘resource matching’ hypothesis as the mechanisms increasing the effectiveness in coping with pain severity as a means toward medication reduction or replacement in a sample of participants with a chronic pain syndrome and to also verify two probable ways of their development (see below) in a healthy sample without chronic pain but who may have experienced moments of occasional acute pain in the past with variabilities in their number, longevity, and intensity (a retrospective lifetime history of episodes of acute pain).

To these aims, participants with fibromyalgia or fibromyalgia syndrome (FMS) were chosen as they are considered to augment or magnify various body sensations, including nociceptive, using a mechanism of hypervigilance or heightened attention to internal events (body and physical processes) by labelling of even non-noxious stimuli, such as tightness or pressure, as painful when compared to other chronic pain groups, such as rheumatic disorders and osteoarthritis^41,42^. Hypervigilance or hypervigilant response style reflects a perceptual style in which the usual cognitive filtering mechanisms related to a deficiency of the inhibitory system, which dampens the response to aversive events, are not fully engaged. Thus, situational distraction coping styles or traits with the habitual distraction behavior, such as EOT, may be such cognitive filtering mechanisms that can regulate pain intensity by capturing or competing for respective cognitive resources in this group of patients^11,43^ and thus may reduce or even replace medications in regular pain control and in mood regulation (*a partial or total ‘medication replacement’* as positive outcomes of the correctly personalized ‘resource matching’ mechanism in coping with pain).

A healthy control group was also recruited to explore a probable origin of personalized ‘resource matching’ mechanisms in contrast to assessing the group difference in measures, as used in regular case–control studies. Previously, it was suggested that in normal or default mode, these evolutionary-developed mechanisms should protect people from intensive cognitive or extensive affective involvement in events (adversities and challenges such as acute pain) accompanied by an over-arousal response, as a kind of anti-stress mechanisms^20^. Thus, in normal participants, either the occasional experience of higher acute pain intensity was expected to initiate more activation of these cognitive distraction mechanisms acquired through *a positive feedback mechanism*, or the innate predisposition to higher activity of these mechanisms should diminish acute pain sensations through *a negative feedforward mechanism*.

## Results

### A group with fibromyalgia (FMS)

**Figure 1:**
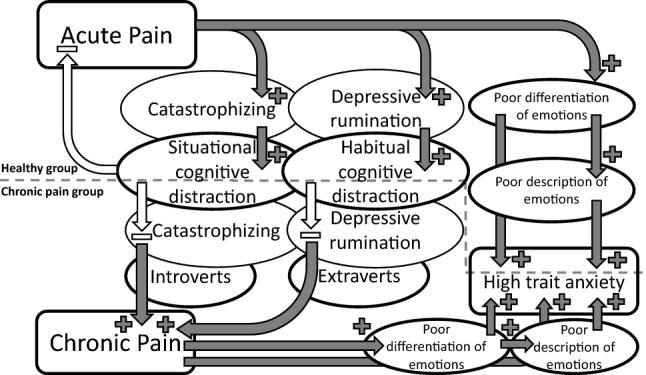
A schema of main direct, interaction, and indirect (mediation and moderated mediation) effects indicated in the study and associated with experienced intensity of occasional acute pain incidences in the healthy group (assessed retrospectively) and chronic pain severity along with drug use in the FMS group. Signs indicate direction of the effects. Experience of acute pain intensity and chronic pain severity were assessed by general and specific to disease scales and inventories such as McGill Pain Questionnaire, Visual Analogue Scale, Body Pain Scale of the Short-Form Health Survey, the Fibromyalgia Impact Questionnaire, analgesics, antidepressants and anxiolytics use. Catastrophizing and situational cognitive distraction were assessed by Coping Strategies Questionnaire. Depressive rumination was assessed by its proxy measure—depression severity obtained from the Beck Depression Inventory. Trait Anxiety was assessed by the State-Trait Anxiety Inventory. Habitual cognitive distraction strategy, capabilities for differentiation, and description of emotions were assessed by the Externally Oriented Thinking, the Difficulty Identifying Feelings, and the Difficulty Describing Feelings subscales of the Toronto Alexithymia Scale, respectively.

Descriptive statistics are presented in Table 1. Correlations between each group of variables are presented in Tables S1–S3 (see Supplementary materials). Figure 1 presents a schema of primary direct, interaction, and indirect (mediation and moderated mediation) effects associated with chronic pain severity in the FMS group.

**Table 1:** Descriptive statistics with means (bootstrap biases) and bootstrap 95% CIs of demographics, psychological, and clinical data of Fibromyalgia patients and Healthy Participants.

Variables	Fibromyalgia Patients	Healthy participants
N	110	60
Age	52.17 (− 0.0033), 50.51 to 53.74	49.32 (− 0.0234), 47.23 to 51.45
Antidepressant	0.65 (− 0.0002), 0.57 to 0.74	0.04 (0.0001), 0.00 to 0.13
Anxiolytic	0.68 (0.0002), 0.59 to 0.77	0.04 (0.0001), 0.00 to 0.13
Non opioid analgesic	0.84 (− 0.0005), 0.77 to 0.90	
Opiate analgesic	0.45 (− 0.0003), 0.35 to 0.54	
Analgesic in total (nonopioids OR opiates)	0.89 (− 0.0001), 0.83 to 0.95	
State anxiety (STAI)	47.99 (− 0.0375), 45.82 to 50.13	34.59 (− 0.0814), 30.63 to 39.40
Trait anxiety (STAI)	63.54 (0.0007), 61.26 to 65.86	42.04 (− 0.0011), 37.54 to 46.74
Depression (BDI)	32.17 (− 0.0223), 29.03 to 35.35	8.81 (0.0030), 5.71 to 12.70
Neuroticism (EPQR-A)	4.78 (− 0.0004), 4.52 to 5.02	2.52 (0.0050), 1.78 to 3.25
Extraversion (EPQR-A)	3.74 (0.0027), 3.46 to 4.03	4.33 (-0.0019), 3.56 to 5.03
Psychoticism (EPQR-A)	2.65 (0.0000), 2.44 to 2.86	2.56 (0.0004), 2.00 to 3.12
Fatigue (FSS)	50.68 (0.0086), 48.52 to 52.79	21.96 (0.0342), 17.31 to 26.87
Sleep satisfaction (OSQS)	2.25 (0.0023), 1.96 to 2.55	6.07 (− 0.0027), 5.58 to 6.53
Insomnia (OSQS)	34.65 (− 0.0128), 32.74 to 36.53	11.89 (0.0020), 10.24 to 14.03
Hypersomnia (OSQS)	6.52 (− 0.0029), 5.72 to 7.30	6.07 (− 0.0027), 5.58 to 6.53
Painful points (MPQ)	30.45 (− 0.0087), 27.40 to 33.72	3.04 (0.0112), 1.97 to 4.42
Words number (MPQ)	31.18 (− 0.0059), 28.74 to 33.65	9.04 (− 0.0039), 6.17 to 12.16
Sensorial pain (MPQ)	42.47 (− 0.0174), 38.23 to 46.93	8.07(− 0.0022), 4.93 to 11.54
Emotional pain (MPQ)	8.66 (0.0001), 7.48 to 9.93	1.44 (0.0018), 0.084 to 2.18
Valorative pain (MPQ)	2.65 (0.0017), 2.34 to 2.98	0.85 (− 0.0001), 0.70 to 0.97
Miscellany pain (MPQ)	12.02 (− 0.0064), 10.61 to 13.48	2.30 (− 0.0060), 1.06 to 3.76
Total pain (MPQ)	65.87(− 0.0215), 59.53 to 72.67	12.67 (− 0.0065), 8.03 to 17.86
Pain intensity (MPQ)	3.51 (− 0.0002), 3.30 to 3.71	1.11 (− 0.0009), 0.92 to 1.30
Pain intensity (VAS)	5.29 (0.0034), 4.83 to 5.78	2.89 (− 0.0039), 2.17 to 3.71
Catastrophizing (CSQ)	21.48 (− 0.0108), 19.71 to 23.15	0.63 (− 0.0021), 0.00 to 1.46
Cognitive distraction (CSQ)	14.85 (0.0050), 13.35 to 16.36	7.63 (− 0.0450), 3.76 to 11.59
Ignoring pain sensations (CSQ)	10.92 (0.0030), 9.75 to 11.97	5.89 (− 0.0218), 2.81 to 9.04
Fibromyalgia impact (FIQ)	69.02 (− 0.0133), 65.91 to 72.00	3.70 (0.0041), 1.05 to 7.03
Body pain (SF-36)	26.39 (0.0058), 25.31 to 27.53	53.21 (0.0000), 48.82 to 56.63
Difficulty Identifying Feelings (TAS-20)	22.36 (− 0.0073), 20.81 to 23.98	12.56 (0.0067), 10.22 to 15.09
Difficulty describing feelings (TAS-20)	16.02 (− 0.0073), 14.92 to 17.09	10.59 (0.0089), 8.41 to 12.95
Externally oriented thinking (TAS-20)	20.49 (− 0.0093), 19.42 to 21.52	15.11 (− 0.0144), 12.81 to 17.38

#### Situational cognitive distraction coping strategy

Two main ‘competition’ and ‘capturing’ mechanisms predicted by the ‘resource matching’ hypothesis and a 'medication replacement' as their outcome were explored by moderation and mediation models examining the relationship between the situational cognitive distraction coping strategy and catastrophizing effects (these two factors were only weakly correlated with each other, see Table S2) on pain severity and medication use in the FMS group. Mediation analysis confirmed the ‘capturing’ mechanism with the 'medication replacement' as its outcome. The use of cognitive distraction determined lower chronic pain severity and avoidance of medication use through decreasing catastrophizing in the FMS group. This mediation effect was found for antidepressant use (higher cognitive distraction → lower catastrophizing → no antidepressant use; B[bootstrap SE] = − 0.018[0.013], bootstrap 95% CIs = − 0.049 to − 0.001) along with all primary pain-related measures obtained by MPQ (except the parameters measuring number of painful sites and PRI-E), VAS, FIQR, and the body pain subscale of the SF-36, e.g., higher cognitive distraction → lower catastrophizing → lower PRI-T, i.e. total pain severity (B[bootstrap SE] = − 0.428[0.232], bootstrap 95% CIs = − 0.949 to − 0.061). In addition, complex serial mediation models with two mediators in chain from cognitive distraction through catastrophizing and pain syndrome severity to medication for pain and mood management were explored and showed the following mediation effects: higher cognitive distraction → lower catastrophizing → lower fibromyalgia impact (from FIQR) → no use of analgesics in total (B[bootstrap SE] = − 0.015[0.011], bootstrap 95% CIs = − 0.044 to − 0.002), → no use of antidepressants (B[bootstrap SE] = − 0.012[0.008], bootstrap 95% CIs = − 0.033 to − 0.001), and → no use of anxiolytics (B[bootstrap SE] = − 0.008[0.006], bootstrap 95% CIs = − 0.025 to − 0.0003).

Moderated mediation analysis confirmed that such personality traits as neuroticism, extraversion, and psychoticism could affect the above cognitive distraction coping effects on pain severity through catastrophizing. This mediation mechanism for the indicated above pain severity measures and for medication use was mainly found in FMS participants with extraversion from mid-range to lower scores (i.e., mainly for introverts) (e.g., for PRI-T, i.e. total pain severity: B[bootstrap SE] = − 0.745[0.293], bootstrap 95% CIs = − 1.383 to − 0.231 at 2.19 [Mean-SD] score of extraversion), with psychoticism from mid-range to lower scores (e.g., for PRI-T, i.e. total pain severity: B[bootstrap SE] = − 0.590[0.408], bootstrap 95% CIs = − 1.621 to − 0.033 at 1.55 [Mean-SD] score of psychoticism), and with neuroticism from mid-range to higher scores (e.g., for PRI-T, i.e. total pain severity: B[bootstrap SE] = − 0.621[0.260], bootstrap 95% CIs = − 1.184 to − 0.163 at 6.00 [Mean + SD] score of neuroticism). The cognitive distraction coping effect through catastrophizing was also found for the body pain subscale of SF-36 only at lower extraversion (B[bootstrap SE] = 0.057[0.031], bootstrap 95% CIs = 0.001 to 0.121 at 2.19 score of extraversion), and only at higher neuroticism (B[bootstrap SE] = 0.048[0.029], bootstrap 95% CIs = 0.001 to 0.114 at 6.00 score of neuroticism). Given the significant correlation between neuroticism and psychoticism (see Table S2), adding psychoticism as a covariate in moderated mediation analyses with neuroticism added no additional value. Thus, only extraversion and neuroticism were found to be unique moderators of the mediation paths from cognitive distraction to pain severity through catastrophizing.

In addition, both mediation and moderated mediation analyses found that higher levels of situational cognitive distraction were associated with lower scores of depressive and trait anxiety symptoms, and fatigue through decreased catastrophizing mainly in participants with lower extraversion and higher neuroticism (data not shown).

#### Habitual cognitive distraction strategy associated with externally oriented thinking facet of alexithymia

Two main ‘competition’ and ‘capturing’ mechanisms predicted by the ‘resource matching’ hypothesis and a 'medication replacement' outcome were explored by moderation and mediation models of the effects on pain severity of the relationship between the EOT facet of alexithymia and severity of depressive symptoms. Mediation analysis confirmed the ‘capturing’ mechanism and the 'medication replacement' outcome. Higher EOT determined lower chronic pain severity and avoidance of medication use through decreased severity of depressive symptoms in the FMS group. This mediation effect was found for antidepressants (higher EOT → lower depressive symptoms → no antidepressant use; B[bootstrap SE] = − 0.029[0.018], bootstrap 95% CIs = − 0.073 to − 0.001) and anxiolytics (higher EOT → lower depressive symptoms → no use of anxiolytics; B[bootstrap SE] = − 0.025[0.017], bootstrap 95% CIs = − 0.066 to − 0.001) along with primary pain-related measures obtained by MPQ (except the parameters measuring number of painful sites and PRI-E), VAS, FIQR, and the body pain subscale of the SF-36 (e.g., higher EOT → lower depressive symptoms → lower PRI-T, i.e. total pain severity; B[bootstrap SE] = − 0.592[0.325], bootstrap 95% CIs = − 1.325 to − 0.047). In addition, complex serial mediation models with two mediators in chain from EOT through depression and pain syndrome severity to pain and mood medication use were explored and showed: higher EOT → lower depressive symptoms → lower fibromyalgia impact (from FIQR) → no use of analgesics in total (B[bootstrap SE] = − 0.025[0.020], bootstrap 95% CIs = − 0.074 to − 0.001) and → no use of antidepressants (B[bootstrap SE] = − 0.018[0.013], bootstrap 95% CIs = − 0.053 to − 0.001).

Moderated mediation analysis confirmed that such personality traits as extraversion, psychoticism, and neuroticism could affect the above EOT effect on pain severity through change in severity of depressive symptoms. This mediation path to the above pain severity measures and for medication use was mainly found in FMS participants with extraversion from mid-range to higher scores (i.e., mainly in extraverts in contrast to the indirect effects of situational cognitive distraction coping mainly found in introverts) (e.g., for PRI-T, i.e. total pain severity: B[bootstrap SE] = − 0.974[0.426], bootstrap 95% CIs = − 1.922 to − 0.263 at 5.28 [Mean + SD] score of extraversion), psychoticism from mid-range to higher scores (i.e., in contrast to indirect effects of situational cognitive distraction coping mainly found at lower psychoticism) (e.g., for PRI-T, i.e. total pain severity: B[bootstrap SE] = − 0.626[0.489], bootstrap 95% CIs = − 1.919 to − 0.080 at 3.74 [Mean + SD] score of psychoticism), and higher neuroticism (i.e., at the same level of neuroticism found for indirect effects of situational cognitive distraction coping) (e.g., for PRI-T, i.e. total pain severity: B[bootstrap SE] = − 0.674[0.383], bootstrap 95% CIs = − 1.556 to − 0.033 at 6.00 [Mean + SD] score of neuroticism). In addition, the EOT effect through severity of depressive symptoms was also found for the cognitive evaluation of pain severity (PRI-E) obtained by MPQ at extraversion and psychoticism from mid-range to higher scores, but in the opposite direction: higher EOT → lower depressive symptoms → higher PRI-E (B[bootstrap SE] = 0.019[0.011], bootstrap 95% CIs = 0.001 to 0.043 at 5.28 [Mean + SD] score of extraversion; B[bootstrap SE] = 0.012[0.011], bootstrap 95% CIs = 0.001 to 0.040 at 3.74 [Mean + SD] score of psychoticism). Though all traits, as assessed by EPQR-A, seemed to be unique moderators of the mediation paths from EOT through depressive symptoms to pain severity, only extraversion and psychoticism uncovered the EOT indirect effects through depressive symptoms, decoupling the evaluative (depicted by PRI-E subscale of MPQ) and affective/sensation (depicted by other subscales of MPQ) pain-related outcomes.

In addition, both mediation and moderated mediation analyses found that higher EOT was associated with decreased scores of catastrophizing, the insomnia subscale of the OQSQ, fatigue, and trait anxiety symptoms but with increased scores on cognitive distraction and the sleep satisfaction subscale of the OQSQ through decreased depressive symptoms mainly in participants with extraversion, psychoticism, and neuroticism from mid-range to higher scores (data not shown).

### A healthy control group without chronic pain

Descriptive statistics are presented in Table 1. Correlations between each group of variables are presented in Tables S3, S4 (see Supplementary materials). Figure 1 presents a schema of main direct, interaction, and indirect (mediation) effects associated with intensity of retrospective acute pain incidences in the healthy group.

#### Situational cognitive distraction coping strategy

Two main ‘competition’ and ‘capturing’ mechanisms predicted by the ‘resource matching’ hypothesis and two ‘positive feedback’ and ‘negative feedforward’ mechanisms of their probable acquired or innate origin were explored by moderation and mediation models of the relationship between the situational cognitive distraction coping strategy and catastrophizing (these two factors were only weakly correlated with each other, see Table S4) on pain severity. Both main mechanisms as well as the ‘positive feedback’ mechanism were confirmed in the healthy group without chronic pain.

Moderation analysis showed that the use of situational cognitive distraction determined experience of lower acute pain intensity, as assessed retrospectively by the PPI subscale of the MPQ, in those healthy participants who had higher than a 15 catastrophizing score, according to the J-N technique (B[Huber-White HC SE] = − 0.005[0.002], t = − 2.129, p = 0.038, bootstrap 95% CIs = − 0.010 to − 0.0003). This interaction effect confirmed the ‘competition’ mechanism predicted by the ‘resource matching’ hypothesis without adjustment for traits assessed by EPQR-A.

Mediation analysis also showed that the use of situational cognitive distraction was associated with a higher impact of retrospective acute pain on subjective well-being through increased catastrophizing in the healthy group, confirming the development of this ‘resource matching’ mechanism related to catastrophizing and cognitive distraction regulations of pain severity through the ‘positive feedback’ process. This mediation effect was found for a primary pain-related measure, the body pain score obtained by SF-36 and two secondary measures (insomnia and sleep satisfaction with the opposite sign): lower SF-36 body pain scores (i.e., higher pain intensity) → higher catastrophizing → higher cognitive distraction (B[bootstrap SE] = − 0.155[0.100], bootstrap 95% CIs = − 0.391 to − 0.006).

#### Habitual cognitive distraction strategy associated with externally oriented thinking facet of alexithymia

Two main ‘competition’ and ‘capturing’ mechanisms predicted by the ‘resource matching’ hypothesis and two ‘positive feedback’ and ‘negative feedforward’ mechanisms of their probable acquired or innate origin were explored by moderation and mediation models of the effects on pain severity from the relationship between the EOT facet of alexithymia and severity of depressive symptoms. Moderation analysis also confirmed the ‘positive feedback’ mechanism of the development of this ‘resource matching’ mechanism related to depressive symptoms and EOT in response to pain experience by showing that higher EOT was determined by higher acute pain intensity, assessed retrospectively, in those healthy participants who had higher severity of depressive symptoms (higher than 18 score according to the J-N technique). This interaction effect was found to be significant for a primary pain-related measure, VAS, and a secondary measure, fatigue without adjustment for traits assessed by EPQR-A (e.g., for VAS: B[Huber-White HC SE] = 0.106[0.038], t = 2.787, p = 0.007, bootstrap 95% CIs = 0.015 to 0.246).

See Supplementary materials for effects of DIF, DDF, and Ignoring Pain Coping Strategy in both groups.

## Discussion

Findings of this cross-sectional study confirmed the hypotheses of indirect and interaction effects of distraction coping strategies on pain severity using resource ‘capturing’ and ‘competition’ mechanisms predicted by the main ‘resource matching’ hypothesis with catastrophizing and depressive symptoms as mediators and moderators, distraction coping strategies as a ‘medication replacement’ alternative in the management of pain and mood, and a ‘positive feedback’ mechanism in the development of the coping strategies. The ‘capturing’ mechanism associated with mediation effects was confirmed in FMS participants, while a ‘competition’ mechanism associated with moderation effects was confirmed in the healthy control group. This study showed that both distraction coping mechanisms, a situational pain-related cognitive distraction (a pain coping style) and a general affect-related habitual cognitive distraction (the EOT trait of alexithymia with high inhibitory control) could be effective in managing (decreasing) pain severity of a chronic pain syndrome, as well as serve as a medication substitute for both somatic and psychological symptoms in chronic pain patients, as was predicted from findings of previous studies^6,22,26,44–46^. Moreover, a mediation model with decreased depressive symptoms as a mediator of the effects of higher EOT on lower pain severity, as indicated in the present study, was concordant with a previous finding of a greater depression severity decrease at follow-up in patients with higher EOT^47^. Some studies showed that, in select cases (e.g., in response to multimodal psychotherapeutic treatment), higher EOT could predict higher depression as a ‘positive feedback’ mechanism (i.e., transferring cognitive resources from EOT style of thinking back to ruminative thinking), and this unfavorable outcome was considered to be related to incorrect selection of a psychological procedure that may cancel the protective EOT effect as a treatment intervention in an environment negative or unfavorable for such patients^20,48–50^. The present findings supported the personalized approach in pain treatment, as was demonstrated in a prospective study with respect to physiological phenotypes^2^, and generalized it to psychological phenotypes. Thus, a personalized approach in selection of correct psychotherapeutic treatment procedures should be central in achieving the desired efficacy of these treatments.

When nociceptive stimuli trigger corresponding central and peripheral arousal changes, they are transferred to central pain sensation, pain feeling, and pain-related negative thinking representations^51^. The main ‘resource matching’ hypothesis suggests that if pain catastrophizing (i.e., disorganized, bizarre, or disoriented thinking) and depressive rumination (negative thinking) are assumed to form the pathways for perpetuating and exacerbating the nociceptive information and thus for contributing to pain severity increases and respective medication overuse, they should be particularly sensitive to both situational (thought-related) and habitual (affect-related) cognitive distraction mechanisms. The distraction of attention from the sources associated with disoriented and negative thoughts is a central coping response of reallocation of cognitive resources for processing non-negative information delivered to the brain. However, these distraction mechanisms may interrupt the perpetuation and exacerbation of the nociceptive stimulation not simply by diverting attention from the main sources of negative experience but through the detachment of the affect-related (ruminative) or psychotic-like (disorganized or catastrophizing) thoughts associated with pain from the physiological resources supporting them, i.e., redirecting respective physiological resources from supporting attention to negative stimulations to preserving attention to neutral information like the contrast in allocation of autonomic resources for environment intake and reject behaviors^19,52^.

**Figure 2:**
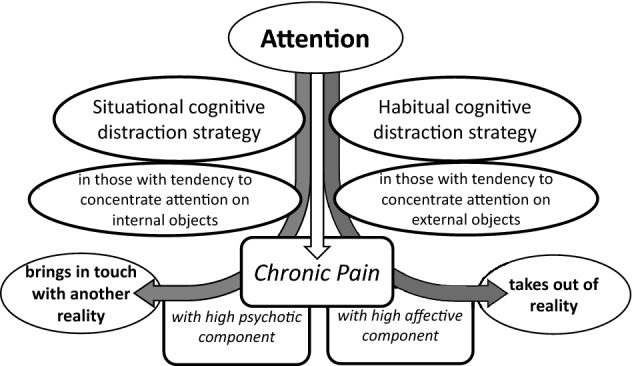
A schema of situational and habitual cognitive distraction mechanisms redirecting attention from the pain experience with a high psychotic-like or high affective component to other dominant, real or unreal events in individuals with tendencies to concentrate attention on internal or external objects, respectively, thus reducing pain severity and need for pain medication.

In correspondence with the ‘resource matching’ hypothesis, the present study extended previous findings that maladaptive thinking-regulation associated with catastrophizing (i.e., psychotic-like) mechanism and maladaptive affect-regulation associated with depression mechanism in coping with pain were not only related to different autonomic regulation of cardiovascular activity (assessed by a modified orthostatic stress test^4^) but also to central pain-regulation (distraction) mechanisms as indicators of different pathogenesis of chronic pain severity in patients with different phenotypes. Accordingly, in the present study, the first (situational coping) mechanism decreasing pain severity and other secondary symptoms of the disease, such as fatigue, through lowering catastrophizing (helplessness and magnification) was found to be mainly effective (i) in FMS participants with extraversion from mid-range to lower scores, i.e., in introverts or those with tendency to concentrate more attention on internal thoughts, excitement-avoidance, and higher sensitivity to stress, and (ii) in FMS participants with higher neuroticism, i.e., those with tendency to be hypervigilant toward somatic sensations and their interpretation as negative or threatening. Thus, more internal resources can be released for favoring this situational distraction mechanism to cope with chronic pain severity in those with higher pain catastrophizing coupled with the predisposition to mid-ranged or higher concentration of attention on internal thoughts and the interpretation of somatic sensations as negative (Figs. 1, 2).

The second (habitual coping) mechanism decreasing pain severity through lowering depressive symptoms including depressive rumination was mainly effective (i) in FMS participants with extraversion from mid-range to higher scores, i.e., in extraverts or those with tendency to concentrate attention on external objects, excitement-seeking, and stress immunity, and (ii) in FMS participants with psychoticism from mid-range to higher scores, i.e., in those with tendency to higher self-centered impulsivity. Thus, more internal resources can be released for favoring this habitual distraction mechanism to cope with chronic pain severity and other secondary symptoms of the disease, such as sleep problems and fatigue, in those with higher depressive rumination coupled with the predisposition to mid-ranged or higher concentration of attention on external objects and self-centered impulsivity (Figs. 1, 2). The habitual distraction mechanism associated with EOT also showed a contrast effect on sensory/affective assessments of pain (with decreased pain severity) and cognitive evaluations of pain (with increased pain severity). This decoupling effect between different components of the pain experience should be taken into consideration as the main by-effect of this trait, associated with the inclination for impression management (i.e., the increase of cognitive evaluations of pain) in communication with other people^19^. Since, this essential attribute of EOT strategy was only found with respect to moderation effects from extraversion and psychoticism, but not from neuroticism, only the former two traits were considered to be important in supporting the EOT indirect effects on pain severity and medication use through lowering depressive symptoms. Thus, if correctly personalized, both distraction mechanisms could regulate severity of a chronic pain syndrome and thus avoid the need for medications to control pain intensity and to regulate mood (Fig. 2). Individuals with mid-range extraversion seem to be sensitive to both kinds of distraction coping strategies. In general, the findings suggest that the effectiveness of these psychological mechanisms could be equivalent in outcomes to effectiveness of non-opioid and opioid analgesics, antidepressants, and anxiolytics.

Some examples of habitual EOT-related distraction strategy could be suggested for psychotherapeutic interventions. They include regular practices of praying as operational or formal rituals or religious meditations without a real attachment to God (i.e., taking out of reality) as presented in some adepts of Christian, Jewish, Muslim, Buddhist, Hindu, and Shamanic worships, other simple spiritual activities and rituals, performance of simple arousing sounds, music, and songs with dominant rhythmic and reduced melodic components as presented in Muslim and African art traditions and in some Western musical art practices (e.g., in jazz)^43,53–56^. Among other examples is a practice of transcendental or mantra meditations, i.e., cognitive activities with creating, writing, and focusing on abstract objects, words, visual images, or concepts, simple arts and crafts, with focusing on endless rhythmic processes such as breathing, repetition of simple sounds or phrases, and general words for a long period of time for replacing thinking and inhibiting memory and sensations associated with actual life events and interests, and directed against a real-life emotional experience and empathy, against own daydreaming and fantasying^45,57–62^.

In contrast, situational cognitive distraction mechanism can be manipulated by mindfulness meditations and practices with preconditioned or motivated focus on a sequence of serially changeable material objects or actions (i.e., bringing in touch with reality) as presented in daily life, such as concentration on watching TV/movies, listening to a radio performance, eating (taste and odor of dishes) or simply focusing on cooking meals, washing dishes, shopping, playing a sad melody or singing sad melodic songs (aloud or in one's mind) as mainly presented in Russian traditional art, playing mental games, complex arts and crafts, walking or being/talking with other people etc., with full awareness of the associated movements, sensations, cognitions, and feelings that may be present during or accompany the events^63–65^. Habitual EOT-related distraction strategies can be based on sustainable motivations approaching intangible matters, such as faith in the God or Samadhi but without affective attachment, through only concentration on a single object or action, such as a prayer or mantra, for the same length of time as in situational cognitive distraction strategies associated with preconditioned or motivated attention not to an intangible goal, but to a sequence of concrete objects or actions. Both techniques can help to distract (disengage) attention from adversities, but differently: one takes the person out of reality in total as associated with the dominant negative event while the other brings the person down to another external reality selected as dominant, not associated with the negative event^43,51,66,67^. As was found in this study, both distraction strategies as psychological ‘opioids’ or the Jean-Jacques Rousseau ‘s ‘opium for soul’^55^ could replace the need for analgesic medications, including opioids, for successful pain management in chronic pain patients.

In the healthy group, a ‘competition’ mechanism as one of predicted by the ‘resource matching’ hypothesis was confirmed by a finding that the use of situational cognitive distraction mechanism determined lower intensity of acute pain sensation during its occasional incidences, as assessed by a number-word designation scale in those participants who had higher catastrophizing strategy. This result corresponded with a previous finding of a greater analgesia in response to acute pain stimulation by distraction behavior in chronic pain patients with higher catastrophizing^7^. Thus, while both distraction strategies were found to help cope with chronic pain, only the situational cognitive distraction mechanism could help in coping with acute pain incidences. A ‘positive feedback’ mechanism was also confirmed in the healthy group by findings that both specific pain- and general affect-related cognitive distraction mechanisms could be acquired through the acute pain experience, but the former mainly at night (as the specific pain distraction mechanism was additionally associated with sleep disturbances) and the latter during daytime (as the general affect-related distraction mechanism was additionally associated with fatigue) through mechanisms connected, respectively, with catastrophizing (through mainly psychotic-like suffering from pain) or depressive ruminations (through mainly affective suffering from pain) (Fig. 1). The ‘positive feedback’ mechanism of the development of the cognitive distraction strategies as an outcome of experience of occasional acute pain incidences was additionally confirmed by the absence of the essential attribute of EOT to decouple somatic/affective sensations from evaluative assessments in these effects^19^. The present findings are concordant with previous studies that showed the association between higher diverting attention and catastrophizing coping strategies and increased pain sensitivity in healthy participants^68^. Thus, the development of cognitive distraction strategies as habitual and conditioned coping styles in healthy participants may be a risk factor for chronification of pain and related disease, because these strategies do not solve the problem but instead distract from it. However, these strategies may help to reduce the experience of stressors, including pain, in both healthy (as a short-term pain relief) and patient (as a long-term pain relief) populations. Thus, the treatment approach for reducing the EOT facet of alexithymia and another situational distraction strategy may be useful for preventing chronic pain development but not for reducing the clinical and economic burdens of already existing chronic pain, as some researchers incorrectly suggest^69^. In the FMS group, ignoring pain coping strategy was found to have a direct negative effect on pain intensity measured by a number-word designation that disappeared in the presence of a situational cognitive distraction strategy indicating the latter as the main coping mechanism in this case (see Supplementary materials).

Findings of the present study also confirmed the importance of alexithymia features assessed separately by EOT, DIF, and DDF scores, rather than the total alexithymia score, as specific factors affecting mind and body regulation mechanisms^39,70^. In contrast to EOT facet, DIF and DDF facets of alexithymia in both the healthy and the FMS groups, mediated severity of primary pain-related measures to higher trait or implicit anxiety symptoms and thus seemed to redirect central regulation of acute and chronic pain sensations to this stable affective temperament (see Supplementary materials and Fig. 1). This mechanism could be evolutionarily developed to restore nociceptive system after habituation or adaption to current painful sensations for sensing new aversive stimuli in the future by transferring the current somatic pain experience to its non-habituated affective component^71^. Thus, although most researchers view alexithymia as a risk factor for pain, the opposite may occur—the experience of stressors, including pain, may reduce the ability to perceive, identify, and/or differentiate emotions as a protective mechanism for inhibiting attention to pain sensation or redirecting its somatic negative experience to the affective system.

In summary, this study confirmed a ‘capturing’ mechanism predicted by the ‘resource matching’ hypothesis with more effective cognitive resources released for coping with the pain experience in chronic pain patients with higher pain catastrophizing or depressive rumination, a ‘competition’ mechanism predicted by the ‘resource matching’ hypothesis with redistribution of restricted physiological resources between the distraction processes and the pain catastrophizing process for coping with acute pain episodes in participants without a habitual pain, and a ‘positive feedback’ mechanism for the development of the distraction coping mechanisms through catastrophizing, such as disorganized thinking, or depressive symptoms, such as ruminative thinking, in response to the acute pain experience in participants without habitual pain. Moreover, patients with a predisposition for concentration of attention on external objects, as well as with excitement-seeking and stress immunity features, were found to effectively use only a habitual affect-related distraction coping mechanism (i.e., EOT component of alexithymia) to capture or relocate internal resources from pain through lowering severity of depressive symptoms (i.e., affective thinking) and thus reducing severity of pain and the need for medications. However, individuals with a predisposition for concentration of attention on internal objects, as well as with excitement-avoidance and stress sensitivity features, were found to effectively use only a ‘conditioned to pain’ distraction coping mechanism to capture or relocate internal resources from pain through lowering catastrophizing (i.e., disorganized thinking) and thus reducing severity of pain and the need for medications. Other components of alexithymia seem to redirect frequent pain sensations to a stable affective trait as a psychological proxy of a high sympathetic arousal phenotype.

### Limitations

Fibromyalgia is considered to be a special syndrome of chronic pain with an augmented or magnified experience of various body sensations, including nociceptive. This likely increases relevance of such distraction coping mechanisms in patients with this syndrome, because they influence its main mechanism of hypervigilance or heightened attention to internal events (body and physical processes) by labelling even non-noxious stimuli as painful. The cross-sectional design of the study with only female participants also limits its inferences. Depressive rumination was indirectly assessed by total BDI-II scores including the symptom as its main factor and thus this substitution was not expected to affect direction and significance of effects very much except effect size. While self-report instruments could be considered as a weakness of the study, multiple measures of the same outcome using these instruments along with the inclusion of medication use in the models as an objective measure of mental and somatic components of pain severity or intensity (confirmed by prescriptions and physicians’ reports) increased the reliability of findings and inferences of the study. To generalize the findings and to confirm the causal pathway inferences, they should be investigated in patients of both sexes with other chronic pain syndromes in studies with a prospective design and with other objective measure of chronic pain severity and related complications, such as fatigue and sleep problems^2,4,12,51,72–74^.

## Conclusion

Habitual hypervigilance or hypervigilant responses to internal or external events may be a cause of chronic pain development in some people predisposed to such a perceptual style with a deficiency of an inhibitory system or cognitive filtering mechanisms, which should normally dampen extensive responses to aversive events. In these cases, conditioned distraction coping styles and traits with the habitual distraction behavior may become such cognitive filtering mechanisms that can decrease intensity of acute pain episodes for healthy individuals or be a treatment choice to replace analgesic and mood regulating medications in patients with chronic pain syndromes. Since, chronic pain and substance abuse conditions are known to share a common cognitive dysfunction, these cognition treatment strategies can also reduce the risk for the development of an opioid use disorder^75,76^. Moreover, a personalized selection of these psychological methods for pain management can reduce reliance on medications for mood and pain control in patients with chronic pain syndromes and thus reduce related complications, such as opioid overdose and premature death. Additional findings in people without habitual or chronic pain showed that these cognitive filtering mechanisms could be developed in response to adversities and challenges, such as occasional acute pain episodes, as a kind of anti-stress resiliency mechanisms^25^.

## Materials and methods

### Participants

One hundred and ten women with fibromyalgia syndrome (FMS) recruited from the Fibromyalgia Association of Jaén (Spain) participated in the study. All of them were examined by a rheumatologist and met the 1990 American College of Rheumatology criteria for FMS^77^. Participants were asked not to consume non-opioids analgesics and/or opioids for 24 h before the study. No special instructions were provided regarding consumption of anxiolytics and antidepressants, which were taken as usual. During the first session, clinical histories, medication use, and socio-demographic data were recorded, and meeting of inclusion/exclusion criteria was confirmed. The control group comprised 60 healthy women recruited from the city population by a flyer. As the main research objective of the study pertained to the analysis of relationships within the FMS group, a smaller control group was proposed to be adequate. The sample sizes were considered to be adequate to control sampling errors in the groups using bootstrap methods for obtaining robust estimates of mediation, moderated mediation, and moderation effects^78–80^. Exclusion criteria for both study groups included the presence of metabolic abnormalities, neurological disorders, drug abuse, and severe somatic (e.g., cancer) or psychiatric (e.g., psychotic) diseases. Participants in the healthy control group were furthermore required not to suffer at the moment from acute or chronic pain of any kind and not to consume any analgesics. All participants provided signed informed consent and were fully debriefed. The study protocol was approved by the Ethics Committee of the University of Jaén. The investigation conforms with the principles outlined in the Declaration of Helsinki. The data that support the findings of this study are openly available in the Open Science Framework and were used in part in another study^11^.

### Psychological assessment

Clinical history and demographic data of all participants were obtained in a semi-structured interview. The Structured Clinical Interview for Axis I Disorders of the Diagnostic and Statistical Manual for Mental Disorders (SCID-I)^81^ was used to diagnose possible mental disorders. In addition, the following self-report questionnaires were administered (values of Cronbach’s α taken from the literature are indicated).

#### Primary pain-related measures

##### McGill Pain Questionnaire (MPQ)^82,83^

This 73-item instrument allows quantification of clinical pain severity and intensity. It includes the parameters of pain location (a number of painful sites), present pain intensity (PPI in number-word designation; 0-none, 1-mild, 2-discomforting, 3-distressing, 4-horrible and 5-excruciating), total number of words chosen (NWC; score range: 0–60), pain quality as Pain Rating Indices (PRI) based on the rank values of the words related to sensory (PRI-S; score range: 0–105), affective (PRI-A; score range: 0–22), evaluative/cognitive (PRI-E; score range: 0–10), miscellaneous (supplementary pain qualities; PRI-M; score range: 0–30) and total (PRI-T; score range: 0–167) pain experiences. Values of Cronbach’s α (0.56 for emotional pain and 0.74 for total pain) and reliability were previously reported^83,84^. The MPQ was presented with the following instructions, differing between the groups; for participants with FMS, the MPQ instructions were presented as a chronic pain assessment instrument: “Indicate your feelings and sensations that you usually associate with your pain”, while for healthy participants, the MPQ was presented as an assessment instrument for retrospective history of occasional acute pain incidences: “To complete this questionnaire I need you to think about infrequent pain that you may have suffered from or about your most common pain problems. Indicate your feelings and sensations that you usually associate with your pain”. In addition, current pain intensity as a whole, if present, was assessed using *a visual analogue scale (VAS)* as a 10 cm horizontal line anchored on the left with "no pain" and on the right with "pain as bad as it could be"^84^. A pain intensity score was obtained by the participant placing a mark to indicate the intensity of present pain. A micrometer was used to measure from the left side of the line to the place marked by the participant. Validity (r = 0.86–0.95), reliability (r = 0.95–0.96), and sensitivity of the VAS as a measure of pain intensity were reported elsewhere^84^. While the PPI and VAS had a correlation of 0.76 for arthritis patients in previous work, their correlation was low and nonsignificant (0.21) for patients with fibromyalgia^84^.

##### The Short-Form Health Survey (SF-36)^85,86^

The SF-36 is one of the instruments most frequently used to assess health-related quality of life and has the advantage of evaluating its various dimensions and components. Only the body pain subscale score (scored via an inverse scale; greater values indicate lower pain) from the SF-36 was utilized for the current study^85,86^. It measures both pain and the interference it produces in usual daily activities. Cronbach´s α value for the body pain subscale of a Spanish version of the SF-36 were previously reported as 0.86^87^.

##### The Fibromyalgia Impact Questionnaire-Revised (FIQR*)*^88^

FIQR is a commonly used instrument in the evaluation of patients with FMS on the domains of function, overall impact, symptoms, including questions on memory, tenderness, balance, and environmental sensitivity. All 21 questions are graded on an 11-point, 0–10 numeric scale with 10 being 'worst' (score range from 0 to 100 with higher value indicating greater impact of the syndrome). The Cronbach’s α for the FIQR was 0.95, with a good correlation with the original FIQ^88^.

##### Medication use

For both groups, information was collected on the current use of pain and mood medications (separately for antidepressants, anxiolytics, nonopioid analgesics, opiates, and analgesics as a combined variable, labelled analgesic in total, which was defined as nonopioid analgesics OR opiates) as an objective proxy measure of mental and somatic components of pain severity or intensity along with the primary measures. Medications were classified based on the following groupings: antidepressants, anxiolytics, non-opioid analgesics, muscle relaxants, complex drug combinations, and opioids (see details in Supplementary materials). As the correct dosage of each medication could not objectively be verified in the study for the assessment of a partial medication replacement or reduced medication use, only a more rigorous dichotomous outcome, use or nonuse of medication, was included in the analysis and further discussed as a total medication replacement outcome. To qualify for the study, participants in the healthy control group were required not to consume any analgesics.

#### Secondary health-related measures

##### The State-Trait Anxiety Inventory (STAI)^89^

This instrument allows assessment of current and habitual anxiety levels (20 items for both the State Anxiety, and Trait Anxiety subscales; 4-point Likert scales from 1 [not all] to 4 [very much so], summed scores ranged from 20 to 80). Values of Cronbach’s α were previously reported as 0.93 for the State Anxiety and 0.87 for Trait Anxiety scales^89^.

##### The Fatigue Severity Scale (FSS)^90,91^

This scale allows assessment of fatigue based on 9 items (7-point Likert scales, summed score range: 9–63), with a reported Cronbach’s α of 0.88^91^.

##### The Oviedo Quality of Sleep Questionnaire (OQSQ)^92^

The OQSQ possesses 15 items scored on a 5-point Likert scale (except for item 1, [subjective sleep satisfaction], which is rated according to a 7-point scale). Indices of the OQSQ used in this study included sleep satisfaction (score range: 1–7), insomnia (score range: 9–45), and hypersomnia (score range: 3–15). A Cronbach’s α of the questionnaire was reported as 0.77^92^.

#### Independent, mediation, and moderation factors

##### The Beck Depression Inventory-II (BDI-II)^93,94^

This 21-item scale was used to assess the severity of symptoms of depression in cognitive-affective and somatic-vegetative domains (4-point Likert scales from 0 to 3, summed scores range: 0–63). Cronbach’s α was previously reported as 0.95^94^. In this study, a greater severity of depressive symptoms assessed by BDI-II was also interpreted as an indicator of greater depressive rumination and its persistence—the main latent factor accounting for the most variance of this inventory’s values in a study of chronic pain patients^16^. Indeed, many previous studies have indicated that repetitive negative thinking associated with rumination was the main vulnerability factor explaining severity of current and past depressive episodes as measured by BDI-II, as well as the main predictor or mediator of future onset of new depressive episodes in response to negative events^95–99^.

##### The Toronto Alexithymia Scale (TAS-20)^100–102^

This instrument measures alexithymia via the three 5-point Likert subscales of Difficulty Identifying Feelings (DIF; score range: 7–35), Difficulty Describing Feelings (DDF; score range: 5–25), and Externally Oriented Thinking (EOT; score range: 8–40). All three subscales were separately used in the current study. Evidence of reliability and factorial validity of the TAS-20 were established, with Cronbach’s αs for the DIF, DDF, and EOT subscales in the Spanish version of the TAS-20 as 0.77, 0.73, and 0.61, respectively^58,100–102^.

##### The Coping Strategies Questionnaire-Revised (CSQ-R)^103,104^

The pain catastrophizing, diverting attention from painful sensations (cognitive distraction), and ignoring pain sensations subscales of this instrument were selected to assess individual disposition to these 3 main coping strategies associated with 3 independent factors (helplessness, diverting attention, and suppression, respectively). Each coping strategy subscale consists of 6 items with 7-point Likert scale from 0 [never] to 6 [always] and score ranges from 0 to 36. Cronbach’s αs for these subscales were reported as 0.78, 0.85, and 0.81, respectively, and were found to be associated with different pain-related outcomes: affective (depression and anxiety), sensorial, and functional, respectively^104^.

Compared to the situational (diverting attention from painful sensations, specifically) and habitual (EOT cognitive style with diverting attention from affective stimuli, in general) cognitive distraction mechanisms, the ignoring pain sensations coping strategy does not explicitly require the participant to reallocate cognitive resources from pain to other objects or events but only to stop (suppress) allocating these resources to pain sensation. Thus, this strategy should also have moderation or mediation relationships with depressive rumination and catastrophizing that affect pain severity, partly sharing the same paths with the cognitive distraction mechanisms. This strategy was explored in the current study to compare the effect of this avoidance or ‘incomplete distraction’ mechanism with the two situational and habitual ‘complete distraction’ mechanisms, and the respective results were presented in Supplementary materials.

##### The Eysenck Personality Questionnaire Revised-Abbreviated (EPQR-A)^105,106^

The EPQR-A is a 24-item questionnaire that is used to measure the personality dimensions of neuroticism (N), extraversion (E), psychoticism (P), and social desirability (Lie scale). Only the first three dimensions were used in the current study with scores ranging between 0 and 6 on all subscales (answer format = YES/NO). Due to its relative brevity, this questionnaire is particularly suited for use in clinical settings and correlates adequately with its well-established longer predecessor, the Eysenck Personality Questionnaire^107^. Cronbach’s αs for N, E, and P scales that were used in the present study were 0.77, 0.82, and 0.52, respectively^105^.

### Statistical analysis

Descriptive and inferential analyses were performed with IBM SPSS Statistics (Version 24.0, Armonk, NY: IBM Corp.). Descriptive statistics were calculated for each variable separately for the FMS and healthy groups, as well as their intercorrelations using Pearson's correlation coefficients. To confirm the main hypotheses, a regression analysis was used to test simple mediation models (i.e., with one mediator; independent factor → mediation factor → dependent variable; cognitive distraction coping style or EOT → catastrophizing or depression → primary pain-related measures and secondary health-related measures) as well as (i) complex mediation models (i.e., with two mediators; independent factor → mediation factor1 → mediation factor2 → dependent variable; cognitive distraction coping style or EOT → catastrophizing or depression → a primary pain-related measure → medication use [YES/NO]), (ii) moderated mediation models (cognitive distraction coping style or EOT → catastrophizing or depression → primary pain severity/intensity and secondary health-related measures at ± 1SD of mean of each trait obtained by EPQR-A), and (iii) moderation models (cognitive distraction_*_catastrophizing or EOT_*_depression → primary pain severity/intensity and secondary health-related measures).

Moderation effects were obtained using SPSS built-in bootstrap procedure. Bias-corrected and accelerated (BCa) bootstrap procedure with 5000 bootstrap samples was used in moderation models to generate non-parametric standard errors and 95% confidence intervals (CIs) of regression coefficients from empirical sampling distribution to avoid misleading inferences (i.e., to balance the risk of Type I and II errors) that are possible during parametric analyses in this relatively small sample^108,109^. In these cases, observed relationships were considered unlikely to be results of chance when values of CIs did not include zero. Bootstrapping is often used as a flexible and robust nonparametric alternative to statistical inference based on parametric assumptions (such as normally distributed errors), because assumptions about distribution can be avoided by this method. The procedure allows assigning measures of accuracy defined in confidence intervals when those assumptions and the stability of the results are in doubt or uncertain, as is often the case in biological and psychological studies. Assumption of homogeneity of variance (homoscedasticity) could have been violated in moderation models and in order to not overestimate the goodness of fit of the moderation models, the Huber-White (heteroscedasticity-consistent, HC) standard errors were used to calculate significance of effects using SPSS command set 'PROCESS'^110^. The Johnson–Neyman (J-N) technique included in the same SPSS macro command set was used to detect regions of significant relationships in the cases of significant moderation (interaction) effects. Values of two-tailed p < 0.05 were regarded as statistically significant in these analyses of moderation models.

Percentile bootstrap procedure with 5000 bootstrap samples was used in mediation and moderated mediation analyses using SPSS command set 'PROCESS' to generate non-parametric 95% confidence intervals (CIs) of regression coefficients from empirical sampling distribution to evaluated the effect for their ‘significance’^110^. As for direct regression models, the observed relationships in these indirect, mediation models were considered with 95% confidence “true” or unlikely due to chance if CIs did not include zero. The bootstrapping technique was found to more accurately capture the shape of the sampling distribution and therefore has greater power to detect mediation^111^.

All parameter estimates were expressed as non-standardized (B) regression coefficients and their standard errors (SE). Since all the above-mentioned analyses were conducted to confirm predictions related to specific hypotheses and following the recommendations outlined by Rothman^112^, no compensations for the number of inferences (i.e., multiple testing correction like the Bonferroni test) were made in either model. Moreover, CIs generated for each effect by the bootstrap procedure (a permutation test or a resampling-based method of inference) guaranteed family-wise type I error control associated with the multiple comparison problem^113^.

## Supplementary Information

Supplementary Information.

## Data Availability

The data that support the findings of this study are openly available in the Open Science Framework at https://osf.io/4wc5n/?view_only=2b0afde14978411085c67ae8b83c53e4. Dual Publication: All the authors gave permission to reuse the data.
