# Supplementary material for: Personalized behavior management as a replacement for medications for pain control and mood regulation
Source: Sci Rep. 2021 Oct 13;11:20297. doi: 10.1038/s41598-021-99803-x (PMC8514566; doi:10.1038/s41598-021-99803-x)
Supplement: Supplementary file 1 — Supplementary Information. [file 41598_2021_99803_MOESM1_ESM.pdf]

Supplementary materials

Title: Personalized behavior management as a replacement for medications for pain control and mood regulation

Short title: Personalizing behavior strategies

Dmitry M. Davydov [*, a, b], MD, PhD, Dr. med. hab.

Carmen M. Galvez-Sánchez [c], PhD

Casandra Isabel Montoro [c], PhD

Cristina Muñoz Ladrón de Guevara [c], MA

Gustavo A. Reyes del Paso [c], PhD

Affiliations

[a] Laboratory of Neuroimmunopathology, Institute of General Pathology and Pathophysiology, Russian Academy of Sciences, Moscow, Russia; Email: d.m.davydov@gmail.com

[b] University of Jaén Hospital, FIBAO, Jaén, Spain; Email: d.m.davydov@gmail.com

[c] Department of Psychology, University of Jaén, Jaén, Spain; Emails: cgalvez@ujaen.es; imontoro@ujaen.es; cmladron@ujaen.es; greyes@ujaen.es

*** Corresponding author information:**

Dmitry M. Davydov, Laboratory of Neuroimmunopathology, Institute of General Pathology and Pathophysiology, Russian Academy of Sciences, 8 Baltiyskaia ul., Moscow, 125315, Russia.

Email: d.m.davydov@gmail.com

Methods

Medication use.

Antidepressants included serotonin–norepinephrine reuptake inhibitors such as Duloxetine, Venlafaxine and Desvenlafaxine, tricyclic antidepressants such as Amitriptyline, selective serotonin reuptake inhibitors such as Fluoxetine, Paroxetine, Escitalopram, Citalopram, and Sertraline, serotonin antagonist and reuptake inhibitors such as Trazodone, and tetracyclic antidepressants such as Mirtazapine, serotonin–dopamine antagonists (atypical antipsychotics) such as Quetiapine. Anxiolytics included benzodiazepines such as Diazepam, Clonazepam, Ketazolam, Medazepam, Lorazepam, Bromazepam, Lormetazepam, Clorazepate and Alprazolam, derivatives of γ-aminobutyric acid (GABA) and $GABA_A$ receptor agonists such as Pregabalin and Zolpidem, and antihistamine drugs such as Hydroxyzine. Non-opioid analgesics included Paracetamol, Metamizol, Niflumic acid, Gabapentin, and nonsteroidal anti-inflammatory drugs (NSAID) such as Ibuprofen, Naproxen, Dexketoprofen, Ketoprofen, Diclofenac, Clonixin Lysine, Meloxicam, Celecoxib. Muscle relaxants included Cyclobenzaprine and Methocarbamol. Complex drug combinations included Naproxen/Esomeprazole magnesium, and complex analgesic and anti-inflammatory liniments and medicated plaster (e.g., with lidocaine). Opioid analgesics included Tramadol (separate and in combination with paracetamol), Morphine and semi-synthetic derivatives of morphine such as Naloxone and Hydromorphone, Codeine in combination with paracetamol/ascorbic acid, Tapentadol, Fentanyl, Buprenorphine (in patches), and Oxycodone. Other medications, such as for high cholesterol, hypertension, thyroid gland dysfunction, as well as anticoagulants and stomach protectors, were also recorded.

Results

A group with fibromyalgia

Difficulty Identifying Feelings and Difficulty Describing Feelings facets of Alexithymia

Mediation analysis with emotional (DIF and DDF) components of alexithymia was also conducted to compare their pain-related links with the distraction mechanisms. These components of alexithymia were moderately positively correlated with each other and very weakly with EOT in this FMS sample (see Table 2S). Severity of the primary pain-related measures obtained by FIQR and MPQ (except the parameters measuring number of painful sites, PRI-E, and PPI) had direct and indirect (through one [DIF] and two-mediators [DIF and DDF]) effects on trait anxiety and depressive symptoms (e.g., higher PRI-A → higher trait anxiety; B[Huber-White HC SE] = 0.890[0.140], bootstrap 95% CIs = 0.597 to 1.178; higher PRI-A → higher DIF → higher trait anxiety; B[bootstrap SE] = 0.102[0.049], bootstrap 95% CIs = 0.022 to 0.212; higher PRI-A → higher DIF → higher DDF → higher trait anxiety; B[bootstrap SE] = 0.124[0.069], bootstrap 95% CIs = 0.023 to 0.291).

In addition, DIF was also found to moderate a positive effect of PPI on trait anxiety and depressive symptoms. According to J-N technique, DIF higher than a score of 31 inhibited the effect of a PPI increase on increase of trait anxiety and depressive symptoms (B[Huber-White HC SE] = -0.217[0.090], t=-2.419, p=0.017, bootstrap 95% CIs = -0.389 to -0.022).

Ignoring Pain Coping Strategy

Two main 'competition' and 'capturing' mechanisms were explored by moderation and mediation models of the relationship between the ignoring pain coping strategy and severity of depressive symptoms on pain severity. Only a direct negative effect of the ignoring pain coping strategy on present pain intensity (PPI) was found for FMS participants (B = -0.044, bootstrap bias[SE, p] = 8.647E-5[0.018, 0.013], bootstrap 95% CIs = -0.077 to -0.012). However, this

effect became likely due to chance when cognitive distraction was added as a covariate (B = -0.039, bootstrap bias[SE, p] = 8.549E-6[0.022, 0.078], bootstrap 95% CIs = -0.082 to 0.006). At the same time, the inclusion of cognitive distraction in the model uncovered a direct negative effect of the ignoring pain coping strategy on the body pain subscale of SF-36. This demonstrated its significant effect on pain aggravation (B = -0.192, bootstrap bias[SE, p] = -0.003[0.082, 0.019], bootstrap 95% CIs = -0.360 to -0.040) with a direct positive effect of cognitive distraction on the body pain subscale (B = 0.243, bootstrap bias[SE, p] = 0.005[0.076, 0.002], bootstrap 95% CIs = 0.101 to 0.410). These findings were consistent with a significant positive correlation between these two coping strategies (Table 2S) and also supported the proposal of the mixed nature of the ignoring pain coping strategy, which included both individuals who actually used the situational cognitive distraction mechanism to help them to cope with pain and those individuals who tried to ignore pain that led to a negative or unfavorable (pain aggravation) outcome. This demonstrated a poor discriminant validity of the scale in this sample. Thus, the ignoring pain coping strategy was found to be less effective in pain management than the cognitive distraction and EOT strategies for these FMS participants.

<u>A healthy control group without chronic pain</u>

Difficulty Identifying Feelings and Difficulty Describing Feelings facets of Alexithymia

Mediation analysis with emotional components (DIF and DDF) of alexithymia were also conducted to compare their pain-related links with the distraction mechanisms. These components of alexithymia were moderately positively correlated with each other and with EOT in this healthy sample (see Table 4S). Severity of the primary pain-related measures obtained by MPQ (except the parameters measuring number of painful sites, PRI-E, and PPI) had indirect (through one [DIF] and two-mediators [DIF and DDF]) effects on trait anxiety (e.g., higher PRI-A → higher DIF → higher trait anxiety; B[bootstrap SE] = 0.924[0.521], bootstrap 95% CIs = 0.164 to 2.168; higher PRI-A → higher DIF → higher DDF → higher trait anxiety; B[bootstrap SE] = 0.587[0.330], bootstrap 95% CIs = 0.100 to 1.341).

Ignoring Pain Coping Strategy

Two main ‘competition’ and ‘capturing’ mechanisms predicted by the main ‘resource matching’ hypothesis and two ‘positive feedback’ and ‘negative feedforward’ mechanisms of their development were explored by examining the relationship between the ignoring pain coping strategy and catastrophizing or depressive symptoms on pain severity, and only the ‘positive feedback’ mechanism using a mediation model was confirmed in the healthy group without habitual pain. This mediation effect was found for a primary pain-related measure, the body pain subscale of the SF-36: higher ignoring pain coping → higher catastrophizing → lower SF-36 body pain scores (i.e., higher pain intensity) (B[bootstrap SE] = -0.145[0.067], bootstrap 95% CIs = -0.287 to -0.028). However, inclusion of situational cognitive distraction and EOT separately as covariates in the model made this mediation effect likely due to chance (B[bootstrap SE] = -0.039[0.063], bootstrap 95% CIs = -0.171 to 0.087 and B[bootstrap SE] = -0.064[0.064], bootstrap 95% CIs = -0.227 to 0.019). This finding was consistent with a significant positive correlation between these coping strategies (Table 4S) and also supported the proposal of the mixed nature of the ignoring pain coping strategy (see above). Thus, similar to findings for the FMS group, the ignoring pain coping strategy was less important than situational cognitive distraction and EOT strategies in coping with the pain experience in this healthy sample.

Discussion

In the FMS participant group, ignoring pain coping strategy was found to have a direct negative effect on pain intensity measured by a number-word designation that disappeared in the presence of a situational cognitive distraction strategy as a coping mechanism. Moreover, after control for the same cognitive distraction coping style, ignoring pain coping strategy was found to have a positive association with pain aggravation indicated by a direct negative effect on the body pain

subscale of the SF-36. These findings were consistent with a positive correlation between the ignoring pain and the cognitive distraction coping strategies and with the proposal of the mixed nature of responses to the ignoring pain coping subscale in this FMS group. Ratings on this subscale could include responses of individuals who actually used the cognitive distraction mechanism, which helped them to cope with pain, and those individuals who tried to ignore (suppress attention to) pain leading to a negative (pain aggravation) outcome. This mixed nature of responses to this subscale demonstrated its poor discriminant validity in this sample of participants with FMS. Thus, in those with FMS, the ignoring pain strategy was considered to be less effective in coping with pain than the two other cognitive distraction strategies.

Findings of the present study also confirmed the importance of alexithymia features assessed separately by EOT, DIF, and DDF scores, rather than the total alexithymia score, as specific factors affecting mind and body regulation mechanisms [1,2]. In contrast to EOT facet, DIF and DDF facets of alexithymia in both the healthy and the FMS groups mediated severity of primary pain-related measures to higher trait or implicit anxiety and thus seemed to redirect central regulation of acute and chronic pain sensations to this stable affective temperament. In the latter group, this mechanism additionally redirected central regulation of pain sensation to depression. Confirmation of this mechanism in two different (pain-free and with chronic pain) populations increased the reliability of the finding. Some authors have suggested that higher levels of alexithymia are associated with both a higher risk of having chronic pain and negative affect disposition (higher anxiety and depression) [3,4]. However, another study showed that a physiological indicator of chronic pain severity, serum C-reactive protein, reduced in parallel with the increase of an affective component of pain in patients with higher alexithymia [5], which supported the switching 'pain sensation-to-pain distress' hypothesis. Thus, high alexithymia associated with these domains, in contrast to the cognitive distraction mechanism associated with EOT, could be a mechanism of transferring occasional phasic responses of physiological systems

to pain sensations to a more stable physiological phenotype (e.g., with low parasympathetic and high sympathetic tone levels) associated with a stable affective temperament or implicit negative affect [6–10]. This mechanism could be evolutionarily developed to restore nociceptive system after habituation to current painful sensations for sensing new aversive stimuli in the future by transferring the current somatic pain experience to its non-habituated affective component [11]. Thus, alexithymia does not predispose to increased sensitivity to new unpleasant stimuli in chronic pain patients [12]. However, improving ability to identify and communicate subjective feelings could have a positive influence on chronic pain perceptions when its somatic component has already been reduced or cancelled [13]. Application of other problem- and emotion-focused coping strategies seem not to be very effective in chronic pain patients with either high EOT or high DIF facets of alexithymia [14]. Thus, although most researchers view alexithymia as a risk factor for pain, the opposite may occur — the experience of stressors, including pain, may reduce the ability to perceive, identify, and/or differentiate emotions as a protective mechanism for inhibiting attention to pain sensation or redirecting its somatic negative experience to the affective system.

References

1. Glaros, A. G. & Lumley, M. A. Alexithymia and pain in temporomandibular disorder. *J. Psychosom. Res.* **59**, 85–88 (2005).
2. Alkan Hartwig, E., Crayen, C., Heuser, I. & Eid, M. It's in the mix: psychological distress differs between combinations of alexithymic facets. *Front. Psychol.* **5**, 1259 (2014).
3. Shibata, M. *et al.* Alexithymia is associated with greater risk of chronic pain and negative affect and with lower life satisfaction in a general population: The Hisayama study. *PLoS One* **9**, e90984 (2014).
4. Di Tella, M. *et al.* Pain experience in Fibromyalgia Syndrome: The role of alexithymia and psychological distress. *J. Affect. Disord.* **208**, 87–93 (2017).
5. Kojima, M. *et al.* Alexithymia, depression, inflammation, and pain in patients with rheumatoid arthritis. *Arthritis Care Res.* **66**, 679–686 (2014).
6. Kano, M., Hamaguchi, T., Itoh, M., Yanai, K. & Fukudo, S. Correlation between alexithymia and hypersensitivity to visceral stimulation in human. *Pain* **132**, 252–263 (2007).
7. Davydov, D. M., Naliboff, B., Shahabi, L. & Shapiro, D. Baroreflex mechanisms in Irritable Bowel Syndrome: Part I. Traditional indices. *Physiol. Behav.* **157**, 102–108 (2016).
8. Davydov, D. M. Cardiac vagal tone as a reliable index of pain chronicity and severity. *Pain* **158**, 2496–2497 (2017).
9. Verkuil, B., Brosschot, J. F. & Thayer, J. F. Cardiac reactivity to and recovery from acute stress: Temporal associations with implicit anxiety. *Int. J. Psychophysiol.* **92**, 85–91 (2014).
10. Schmukle, S. C. & Egloff, B. Does the implicit association test for assessing anxiety measure trait and state variance? *Eur. J. Pers.* **18**, 483–494 (2004).
11. Maeoka, H., Hiyamizu, M., Matsuo, A. & Morioka, S. The influence of repeated pain

stimulation on the emotional aspect of pain: A preliminary study in healthy volunteers. *J. Pain Res.* **8**, 431–436 (2015).

12. Huber, A., Suman, A. L., Biasi, G. & Carli, G. Alexithymia in fibromyalgia syndrome: associations with ongoing pain, experimental pain sensitivity and illness behavior. *J. Psychosom. Res.* **66**, 425–33 (2009).
13. Tulipani, C. *et al.* Alexithymia and cancer pain: The effect of psychological intervention. *Psychother. Psychosom.* **79**, 156–163 (2010).
14. Di Tella, M. *et al.* Coping strategies and perceived social support in fibromyalgia syndrome: Relationship with alexithymia. *Scand. J. Psychol.* **59**, 167–176 (2018).

Table 1S. Correlations (Pearson Coefficient (bootstrap bias) bootstrap 95% CIs) in participants with Fibromyalgia.

	Antidepressant	Anxiolytic	Non opioid analgesic	Opiate analgesic	Analgesic	Fatigue	Fibromyalgia Impact	Body Pain	Sleep satisfaction	Insomnia	Hypersomnia	Painful points	Words number	Sensorial Pain	Emotional Pain	Valorative Pain	Miscellany Pain	Total Pain	Pain Intensity (MPQ)	Pain Intensity (VAS)
Antidepressant		.571* (.001) .40 to .72	.195+ (-.002) -.002 to .39	.382* (.002) .22 to .54	.359* (-.002) .18 to .52	.250* (-.001) .05 to .43	.402* (.000) .24 to .55	-.302* (-.001) -.47 to -.12	-.180 (-.002) -.37 to .01	.201+ (.002) .02 to .38	-.103 (.000) -.29 to .08	-.010 (.006) -.21 to .21	.233+ (.000) .06 to .40	.175 (.000) -.01 to .34	.236+ (-.001) .06 to .40	-.084 (-.008) -.29 to .08	.176 (-.001) -.01 to .34	.194+ (-.001) .02 to .36	.181 (.000) .004 to .36	.330* (.000) .19 to .46
Anxiolytic	.571* (.001) .40 to .72		.331* (-.002) .13 to .52	.259* (.002) .08 to .43	.387* (-.003) .19 to .56	.098 (.000) -.10 to .29	.318* (.001) .14 to .48	-.101 (-.004) -30 to .08	-.180 (.000) -.38 to .01	.105 (.001) -.07 to .29	-.112 (.001) -.31 to .08	.077 (.007) -.13 to .30	.062 (-.001) -.12 to .23	-.007 (-.001) -.20 to .18	.156 (.000) -.03 to .32	-.016 (-.005) -.21 to .15	.037 (-.002) -.16 to .21	.009 (-.001) -.19 to .19	104 (.000) -.07 to .29	153 (.000) -.01 to .31
Non opioid analgesic	.195+ (-.002) -.002 to .39	.331* (-.002) .13 to .52		.100 (-.002) -.09 to .27	.791* (-.004) .63 to .93	-.034 (.001) -.21 to .16	.222+ (.000) .06 to .39	-.212+ (.005) -.43 to .03	-.088 (.000) -.32 to .14	.120 (.001) -.10 to .33	-.039 (.002) -.24 to .16	-.036 (.010) -.27 to .19	.091 (.000) -.09 to .26	.084 (.001) -.11 to .26	.061 (.002) -.13 to .24	.153 (.001) .02 to .28	.133 (.000) -.04 to .29	.074 (.002) -.14 to .26	.049 (.001) -.15 to .24	.043 (.000) -.14 to .22
Opiate analgesic	.382* (.002) .22 to .54	.259* (.002) .08 to .43	.100 (-.002) -.09 to .27		.314* (-.002) .22 to .41	-.075 (.002) -.26 to .11	.206+ (.002) .03 to .38	-.122 (.001) -.29 to .07	-.129 (.001) -.31 to .06	.243+ (-.002) .07 to .40	-.006 (-.001) -.20 to .18	-.094 (.002) -.26 to .09	.025 (.001) -.15 to .21	-.007 (.002) -.18 to .17	.110 (.001) -.07 to .30	.004 (-.003) -.19 to .19	-.042 (.000) -.22 to .14	.023 (.000) -.16 to .20	.068 (-.003) -.12 to .25	.118 (-.001) -.07 to .31
Analgesic	.359* (-.002) .18 to .52	.387* (-.003) .19 to .56	.791* (-.004). 63 to .93	.314* (-.002) .22 to .41		.059 (.001) -.13 to .27	.312* (.000) .15 to .47	-.312* (.007) -.54 to -.04	-.132 (.001) -.34 to .07	.163 (-.001) -.04 to .36	-.111 (.001) -.31 to .08	-.108 (.010) -.37 to .14	.204+ (.000) .04 to .35	.183 (.001) .003 to .34	.154 (.001) -.02 to .30	.030 (-.002) -.11 to .15	.206+ (.000) .05 to .34	.196+ (.000) .03 to .34	.083 (.000) -.14 to .29	.173 (.000) .03 to .30
State Anxiety	.156 (.000) -.03 to .33	.200+ (-.001) .01 to .37	.002 (.000) -.15 to .15	.194+ (.002) .02 to .37	.000 (.001) -.15 to .15	.151 (-.001) -.03 to .33	.156 (.000) -.04 to .35	.157 (.003) -.01 to .34	-.144 (.001) -.32 to .04	-.027 (.000) -.22 to .17	.162 (.000) -.02 to .34	.175 (-.001) .002 to .35	-.022 (.001) -.21 to .18	-.007 (.002) -.21 to .20	.021 (.001) -.18 to .23	.094 (.007) -.15 to .35	-.021 (.000) -.22 to .18	.006 (.000) -.20 to .21	.241+ (-.003) .08 to .39	-.091 (.000) -.26 to .09
Trait Anxiety	.418* (-.001) .26 to .56	.324* (-.002) .14 to .49	.033 (.001) -.16 to .22	.164 (.000) -.02 to .33	.127 (-.001) -.07 to .31	.317* (.000) .14 to .48	.449* (-.001) .30 to .59	-.419* (-.004) -.55 to -.29	-.394* (-.003) -.57 to -.22	.443* (.002) .27 to .61	-.180 (.000) -.36 to .002	.093 (.003) -.07 to .27	.511* (-.001) .38 to .62	.363* (-.001) .21 to .50	.564* (-.001) .42 to .69	-.222+ (-.006) -.42 to -.04	.377* (-.002) .23 to .51	.397* (.000) .24 to .54	.385* (.002) .22 to .54	.585* (-.001) .47 to .69
Depression	.311* (-.002) .15 to .46	.247* (-.001) .08 to .40	.056 (.000) -.11 to .22	.135 (-.001) -.05 to .32	.114 (-.001) -.04 to .26	.318* (.000) .14 to .48	.531* (-.002) .39 to .65	-.488* (-.002) -.62 to -.34	-.440* (-.004) -.60 to -.28	.487* (.001) .34 to .63	-.137 (.001) -.31 to .04	.122 (.005) -.07 to .32	.535* (-.001) .40 to .66	.376* (-.001) .21 to .53	.629* (-.001) .51 to .73	-.214+ (-.004) -.38 to -.07	.370* (-.002) .21 to .52	.424* (-.001) .27 to .57	.395* (.003) .23 to .56	.643* (-.001) .53 to .74
Neuroticism	.127 (-.003) -.08 to .32	.244+ (-.002) .04 to .43	.058 (.001) -.14 to .27	-.018 (-.002) -.21 to .16	.075 (-.002) -.13 to .30	.183 (-.006) -.06 to .40	.168 (-.001) -.02 to .35	.013 (-.003) -.17 to .18	-.187 (.000) -.37 to .01	.173 (.002) -.001 to .35	-.001 (-.001) -.18 to .16	.150 (-.001) -.04 to .33	.202+ (-.004) .02 to .36	.137 (-.003) -.05 to .29	.281* (-.003) .12 to .42	-.051 (-.004) -.21 to .10	.137 (-.003) -.03 to .29	.148 (-.003) -.04 to .31	.141 (.001) -.04 to .32	.139 (-.001) -.02 to .29
Extraversion	-.199+ (.002) -.36 to -.03	-.167 (.000) -.34 to .01	-.028 (-.001) -.21 to .16	-.120 (-.001) -.32 to .07	-.079 (.001) -.24 to .08	-.089 (.000) -.26 to .09	-.002 (-.001) -.17 to .16	-.105 (-.005) -.29 to .07	.084 (-.004) -.11 to .26	.016 (.001) -.19 to .23	-.092 (-.003) -.28 to .08	.087 (.006) -.08 to .28	-.013 (.002) -.22 to .19	-.049 (.001) -.26 to .16	-.255* (.002) -.44 to -.05	-.067 (-.007) -.27 to .10	.007 (.000) -.22 to .22	-.099 (.002) -.31 to .11	-.077 (.001) -.27 to .13	.100 (.001) -.12 to .31
Psychoticism	.079 (-.003) -.10 to .24	.082 (-.003) -.10 to .25	.059 (-.001) -.10 to .22	.123 (-.001) -.07 to .31	.020 (-.003) -.13 to .16	.158 (-.002) -.03 to .34	.118 (-.003) -.07 to .30	.053 (.001) -.12 to .23	.035 (-.002) -.19 to .26	.016 (.001) -.19 to .23	.038 (-.001) -.15 to .23	.216+ (-.006) -.01 to .42	.216+ (-.002) .02 to .39	.261* (-.002) .08 to .42	.135 (.000) -.08 to .33	.157 (.002) .01 to .31	.293* (-.001) .10 to .47	.268* (-.001) .09 to .42	.189+ (-.004) -.004 to .36	.114 (-.007) -.11 to .31
Catastrophizing	.267* (-.001) .10 to .43	.210+ (.002) .03 to .38	.010 (.000) -.16 to .18	.078 (.001) -.11 to .27	.107 (.001) -.05 to .24	.244+ (.001) .08 to .41	.469* (-.001) .33 to .59	-.224+ (-.001) -.38 to -.06	-.276* (-.004) -.49 to -.06	.280* (.000) .07 to .47	-.165 (-.001) -.33 to .000	.057 (.002) -.08 to .20	.483* (-.002) .32 to .63	.337* (-.002) .14 to .51	.573* (-.002) .44 to .70	-.181 (-.002) -.34 to -.03	.302* (-.003) .13 to .47	.371* (-.002) .20 to .53	.321* (.000) .13 to .50	.478* (-.001) .34 to .61
Cognitive distraction	-.198+ (.002) -.37 to -.02	-.216+ (.002) -.38 to -.03	-.056 (-.003) -.26 to .14	-.018 (.003) -.20 to .18	-.071 (.000) -.21 to .07	-.196+ (-.003) -.37 to -.02	-.262* (-.002) -.43 to -.09	.241+ (.003) .08 to .40	.266* (-.002) .04 to .47	-.331* (.001) -.51 to -.14	.185 (-.002) .02 to .35	-.150 (-.002) -.33 to .04	-.119 (-.001) -.29 to .06	-.058 (-.002) -.25 to .13	-.293* (.001) -.45 to -.13	.064 (.003) -.10 to .25	-.054 (-.002) -.22 to .12	-.088 (-.002) -.27 to .09	-.156 (-.001) -.34 to .03	-.249* (.001) -.40 to -.09
Ignoring pain sensations	-.069 (.002) -.24 to .11	-.046 (.000) -.23 to .14	.104 (-.001) -.08 to .28	.009 (.002) -.18 to .20	-.015 (.002) -.14 to .13	-.201+ (.001) -.37 to -.02	-.002 (.001) -.17 to .17	-.021 (-.002) -.18 to .13	.073 (-.001) -.12 to .25	-.017 (.002) -.19 to .17	-.095 (-.003) -.28 to .08	-.077 (.005) -.23 to .10	.025 (.001) -.15 to .20	-.051 (.000) -.23 to .13	-.158 (.001) -.34 to .03	.062 (-.005) -.17 to .25	.065 (.001) -.12 to .24	-.049 (.001) -.22 to .12	-.235+ (.002) -.41 to -.04	-.104 (.004) -.30 to .11
Difficulty Identifying Feelings	.232+ (-.001) .07 to .40	133 (-.001) -.05 to .31	-.094 (-.002) -.30 to .12	.184 (.001) -.002 to .37	.077 (-.002) -.14 to .28	.274* (.000) .10 to .43	.369* (-.002) .19 to.53	-.223+ (.001) -.39 to -.05	-.176 (-.002) -.37 to .02	.195+ (.000) .01 to .38	-.032 (-.001) -.22 to .15	.098 (-.001) -.06 to .24	.380* (-.001) .22 to .52	.299* (-.001) .13 to .45	.348* (-.001) .16 to .52	-.026 (-.001) -.22 to .17	.319* (-.002) .15 to .47	.334* (-.001) .17 to .48	.100 (.001) -.09 to .28	.204+ (-.002) .004 to .39
Difficulty Describing Feelings	.274* (-.001) .11 to .44	209+ (.000) .03 to .40	.031 (-.003) -.17 to .23	.127 (.001) -.06 to .31	.117 (-.001) -.08 to .31	.137 (.001) -.04 to .31	.252* (.001) .08 to .42	-.086 (-.001) -.25 to .07	-.121 (.001) -.32 to .08	.115 (.000) -.09 to .32	-.139 (.000) -.34 to .06	.091 (-.001) -.07 to .25	.233+ (.000) .05 to .41	.197+ (.000) .01 to .38	.273* (.000) .08 to .45	.084 (-.006) -.12 to .26	.208+ (-.001) .01 to .39	.207+ (.001) .004 to .40	.007 (-.001) -.19 to .21	115 (-.001) -.10 to .32
Externally Oriented Thinking	-.035 (-.002) -.21 to .13	.059 (-.002) -.12 to .23	-.001 (.000) -.17 to .17	.025 (.000) -.16 to .20	-.021 (.000) -.21 to .17	.088 (-.003) -.10 to .26	.007 (-.002) -.19 to .20	.190+ (.004) .03 to .37	.045 (.006) -.15 to .25	-.100 (-.003) -.32 to .11	.028 (.003) -.14 to .21	.149 (-.010) -.08 to .34	-.109 (-.002) -.31 to .09	-.042 (-.002) -.24 to .15	-.039 (-.003) -.25 to .17	.223+ (.003) .05 to .39	-.032 (-.001) -.24 to .16	-.040 (-.002) -.24 to .15	-.073 (-.004) -.28 to .12.	-.160 (-.006) -.42 to .10
Fatigue	.250* (-.001) .05 to .43	.098 (.000) -.10 to .29	-.034 (.001) -.21 to .16	-.075 (.002) -.26 to .11	.059 (.001) -.13 to .27		.400* (.001) .19 to .59	-.262* (.002) -.45 to -.05	-.260* (.000) -.43 to -.08	.248* (.001) .06 to .43	.164 (-.001) .01 to .31	.425* (.002) .29 to .55	.314* (-.002) .13 to .47	.217+ (-.002) .02 to .39	.268* (-.001) .11 to .41	-.015 (-.001) -.16 to .12	.184 (-.002) -.01 to .35	.229+ (-.002) .04 to .40	.262* (.000) .08 to .43	.249* (.000) .10 to .39
Fibromyalgia Impact	.402* (.000) .24 to .55	.318* (.001) .14 to .48	.222+ (.000) .06 to .39	.206+ (.002) .03 to .38	.312* (.000) .15 to .47	.400* (.001) .19 to .59		-.396* (-.002) -.53 to -.25	-.318* (-.002) -.51 to -.11	.388* (.002) .20 to .57	-.153 (.001) -.31 to .01	.098 (.008) -.12 to .33	.358* (-.001) .16 to .53	.255* (.000) .05 to .45	.305* (.001) .14 to .46	-.180 (-.006) -.39 to -.01	.271* (-.002) .06 to .46	.266* (.001) .07 to .46	.103 (.000) -.08 to .29	.374* (-.002) .23 to .51
Body Pain	-.302* (-.001) -.47 to -.12	-.101 (-.004) -30 to .08	-.212+ (.005) -.43 to .03	-.122 (.001) --.29 to .07	-.312* (.007) -.54 to -.04	-.262* (.002) -.45 to -.05	-.396* (-.002) -.53 to -.25		.212+ (.010) .03 to .43	-.388* (-.009) -.59 to -.21	.149 (.002) -.02 to .34	.137 (-.022) -.18 to .42	-.448* (-.003) -.57 to -.33	-.369* (-.002) -.49 to -.23	-.351* (-.001) -.47 to -.23	.130 (.011) -.02 to .33	-.402* (-.003) -.51 to -.30	-.386* (-.002) -.51 to -.26	-.227+ (-.002) -.41 to -.05	-.489* (-.004) -.60 to -.38
Sleep satisfaction	-.180 (-.002) -.37 to .01	-.180 (.000) -.38 to .01	-.088 (.000) -.32 to .14	-.129 (.001) -.31 to .06	-.132 (.001) -.34 to .07	-.260* (.000) -.43 to -.08	-.318* (-.002) -.51 to -.11	.212+ (.010) .03 to .43		-.703* (.002) -.82 to -.56	-.045 (.002) -.20 to .12	-.224+ (-.002) -.37 to -.06	-.332* (-.003) -.51 to -.16	-.239+ (-.003) -.42 to -.04	-.348* (.001) -.49 to -.20	.139 (.003) -.04 to .32	-.276* (-.003) -.45 to -.09	-.281* (-.002) -.45 to -.11	-.270* (-.002) -.45 to -09	-.379* (-.001) -.52 to -.23
Insomnia	.201+ (.002) .02 to .38	.105 (.001) -.07 to .29	.120 (.001) -.10 to .33	.243+ (-.002) .07 to .40	.163 (-.001) -.04 to .36	.248* (.001) .06 to .43	.388* (.002) .20 to .57	-.388* (-.009) -.59 to -.21	-.703* (.002) -.82 to -.56		-.047 (-.001) -.22 to .11	.173 (.001) .01 to .32	.361* (.001) .15 to .56	.198+ (.002) -.03 to .41	.293* (-.002) .09 to .47	-.172 (-.010) -.42 to .02	.233+ (.001) .01 to .44	.227+ (.001) .01 to .43	.155 (.001) -.04 to .36	.461* (.000) .29 to .61
Hypersomnia	-.103 (.000) -.29 to .08	-.112 (.001) -.31 to .08	-.039 (.002) -.24 to .16	-.006 (-.001) -.20 to .18	-.111 (.001) -.31 to .08	.164 (-.001) .01 to .31	-.153 (.001) -.31 to .01	.149 (.002) -.02 to .34	-.045 (.002) -.20 to .12	-.047 (-.001) -.22 to .11		-.197+ (-.005) -.03 to .39	.033 (-.003) -.17 to .22	.103 (-.003) -.12 to .31	-.029 (-.001) -.23 to .17	.342* (.003) .13 to .54	.011 (-.003) -.21 to .23	.101 (-.005) -.13 to .31	.070 (-.003) -.13 to .26	-.209+ (-.001) -.39 to -.02
Painful points	-.010 (.006) -.21 to .21	.077 (.007) -.13 to .30	-.036 (.010) -.27 to .19	-.094 (.002) -.26 to .09	-.108 (.010) -.37 to .14	.425* (.002) .29 to .55	.098 (.008) -.12 to .33	.137 (-.022) -.18 to .42	-.224+ (-.002) -.37 to -.06	.173 (.001) .01 to .32	-.197+ (-.005) -.03 to .39		.125 (.004) -.03 to .30	-.011 (.003) -.17 to .17	.050 (.005) -.12 to .25	.192+ (.006) .09 to .31	-.047 (.006) -.21 to .16	-.008 (.004) -.17 to .18	.275* (.003) .15 to .41	.109 (.003) -.06 to .30
Words number	.233+ (.000) .06 to .40	.062 (-.001) -.12 to .23	.091 (.000) -.09 to .26	.025 (.001) -.15 to .21	.204+ (.000) .04 to .35	.314* (-.002) .13 to .47	.358* (-.001) .16 to .53	-.448* (-.003) -.57 to -.33	-.332* (-.003) -.51 to -.16	.361* (.001) .15 to .56	.033 (-.003) -.17 to .22	.125 (.004) -.03 to .30		.880* (-.001) .83 to .92	.727* (-.001) .62 to .81	-.153 (-.004) -.33 to .01	.838* (-.001) .77 to .89	.901* (.000) .86 to .94	.387* (.000) .19 to .56	.608* (-.001) .48 to .72
Sensorial Pain	.175 (.000) -.01 to .34	-.007 (-.001) -.20 to .18	.084 (.001) -.11 to .26	-.007 (.002) -.18 to .17	.183 (.001) .003 to .34	.217+ (-.002) .02 to .39	.255* (.000) .05 to .45	-.369* (-.002) -.49 to -.23	-.239+ (-.003) -.42 to -.04	.198+ (.002) -.03 to .41	.103 (-.003) -.12 to .31	-.011 (.003) -.17 to .17	.880* (-.001) .83 to .92		.616* (.000) .45 to .75	-.079 (-.001) -.26 to .10	.861* (-.001) .80 to .91	.977* (.000) .97 to .99	.345* (.002) .13 to .55	.457* (-.001) .29 to .61
Emotional Pain	.236+ (-.001) .06 to .40	.156 (.000) -.03 to .32	.061 (.002) -.13 to .24	.110 (.001) -.07 to .30	.154 (.001) -.02 to .30	.268* (-.001) .11 to .41	.305* (.001) .14 to .46	-.351* (-.001) -.47 to -.23	-.348* (.001) -.49 to -.20	.293* (-.002) .09 to .47	-.029 (-.001) -.23 to .17	.050 (.005) -.12 to .25	.727* (-.001) .62 to .81	.616* (.000) .45 to .75		-.063 (.002) -.26 to .16	.568* (-.001) .41 to .71	.715* (.000) .59 to .82	.473* (.000) .32 to .61	.597* (-.001) .46 to .72
Valorative Pain	-.084 (-.008) -.29 to .08	-.016 (-.005) -.21 to .15	.153 (.001) .02 to .28	.004 (-.003) -.19 to .19	.030 (-.002) -.11 to .15	-.015 (-.001) -.16 to .12	-.180 (-.006) -.39 to -.01	.130 (.011) -.02 to .33	.139 (.003) -.04 to .32	-.172 (-.010) -.42 to .02	.342* (.003) .13 to .54	.192+ (.006) .09 to .31	-.153 (-.004) -.33 to .01	-.079 (-.001) -.26 to .10	-.063 (.002) -.26 to .16		-.073 (.000) -.26 to .11	-.059 (-.001) -.24 to .13	.009 (.006) -.19 to .25	-.362* (-.003) -.48 to -.24
Miscellany Pain	.176 (-.001) -.01 to .34	.037 (-.002) -.16 to .21	.133 (.000) -.04 to .29	-.042 (.000) -.22 to .14	.206+ (.000) .05 to .34	.184 (-.002) -.01 to .35	.271* (-.002) .06 to .46	-.402* (-.003) -.51 to -.30	-.276* (-.003) -.45 to -.09	.233+ (.001) .01 to .44	.011 (-.003) -.21 to .23	-.047 (.006) -.21 to .16	.838* (-.001) .77 to .89	.861* (-.001) .80 to .91	.568* (-.001) .41 to .71	-.073 (.000) -.26 to .11		.894* (.000) .84 to .94	.355* (.000) .15 to .54	.485* (-.003) .31 to .64
Total Pain	.194+ (-.001) .02 to .36	.009 (-.001) -.19 to .19	.074 (.002) -.14 to .26	.023 (.000) -.16 to .20	.196+ (.000) .03 to .34	.229+ (-.002) .04 to .40	.266* (.001) .07 to .46	-.386* (-.002) -.51 to -.26	-.281* (-.002) -.45 to -.11	.227+ (.001) .01 to .43	.101 (-.005) -.13 to .31	-.008 (.004) -.17 to .18	.901* (.000) .86 to .94	.977* (.000) .97 to .99	.715* (.000) .59 to .82	-.059 (-.001) -.24 to .13	.894* (.000) .84 to .94		.385* (.001) .19 to .57	.496* (.000) .33 to .65
Pain Intensity (MPQ)	.181 (.000) .004 to .36	104 (.000) -.07 to .29	.049 (.001) -.15 to .24	.068 (-.003) -.12 to .25	.083 (.000) -.14 to .29	.262* (.000) .08 to .43	.103 (.000) -.08 to .29	-.227+ (-.002) -.41 to -.05	-.270* (-.002) -.45 to -09	.155 (.001) -.04 to .36	.070 (-.003) -.13 to .26	.275* (.003) .15 to .41	.387* (.000) .19 to .56	.345* (.002) .13 to .55	.473* (.000) .32 to .61	.009 (.006) -.19 to .25	.355* (.000) .15 to .54	.385* (.001) .19 to .57		.606* (-.001) .48 to .71
Pain Intensity (VAS)	.330* (.000) .19 to .46	153 (.000) -.01 to .31	.043 (.000) -.14 to .22	.118 (-.001) -.07 to .31	.173 (.000) .03 to .30	.249* (.000) .10 to .39	.374* (-.002) .23 to .51	-.489* (-.004) -.60 to -.38	-.379* (-.001) -.52 to -.23	.461* (.000) .29 to .61	-.209+ (-.001) -.39 to -.02	.109 (.003) -.06 to .30	.608* (-.001) .48 to .72	.457* (-.001) .29 to .61	.597* (-.001) .46 to .72	-.362* (-.003) -.48 to -.24	.485* (-.003) .31 to .64	.496* (.000) .33 to .65	.606* (-.001) .48 to .71	

Note: + $p < 0.05$, * $p < 0.01$, two-tailed test.

Table 2S. Correlations (Pearson Coefficient (bootstrap bias) bootstrap 95% CIs) in participants with Fibromyalgia.

Variables	State Anxiety	Trait Anxiety	Depression	Neuroticism	Extraversion	Psychoticism	Catastrophizing	Cognitive distraction	Ignoring pain sensations	Difficulty Identifying Feelings	Difficulty Describing Feelings	Externally Oriented Thinking
Antidepressant	.156 (.000) -.03 to .33	.418* (-.001) .26 to .56	.311* (-.002) .15 to .46	.127 (-.003) -.08 to .32	-.199+ (.002) -.36 to -.03	.079 (-.003) -.10 to .24	.267* (-.001) .10 to .43	-.198+ (.002) -.37 to -.02	-.069 (.002) -.24 to .11	.232+ (-.001) .07 to .40	.274* (-.001) .11 to .44	-.035 (-.002) -.21 to .13
Anxiolytic	.200+ (-.001) .01 to .37	.324* (-.002) .14 to .49	.247* (-.001) .08 to .40	.244+ (-.002) .04 to .43	-.167 (.000) -.34 to .01	.082 (-.003) -.10 to .25	.210+ (.002) .03 to .38	-.216+ (.002) -.38 to -.03	-.046 (.000) -.23 to .14	133 (-.001) -.05 to .31	209+ (.000) -.03 to .40	.059 (-.002) -.12 to .23
Non opioid analgesic	.002 (.000) -.15 to .15	.033 (.001) -.16 to .22	.056 (.000) -.11 to .22	.058 (.001) -.14 to .27	-.028 (-.001) -.21 to .16	.059 (-.001) -.10 to .22	.010 (.000) -.16 to .18	-.056 (-.003) -.26 to .14	.104 (-.001) -.08 to .28	-.094 (-.002) -.30 to .12	.031 (-.003) -.17 to .23	-.001 (.000) -.17 to .17
Opiate analgesic	.194+ (.002) .02 to .37	.164 (.000) -.02 to .33	.135 (-.001) -.05 to .32	-.018 (-.002) -.21 to .16	-.120 (-.001) -.32 to .07	.123 (-.001) -.07 to .31	.078 (.001) -.11 to .27	-.018 (.003) -.20 to .18	.009 (.002) -.18 to .20	.184 (.001) -.002 to .37	.127 (.001) -.06 to .31	.025 (.000) -.16 to .20
Analgesic	.000 (.001) -.15 to .15	.127 (-.001) -.07 to .31	.114 (-.001)-.04 to .26	.075 (-.002) -.13 to .30	-.079 (.001) -.24 to .08	.020 (-.003) -.13 to .16	.107 (.001) -.05 to .24	-.071 (.000) -.21 to .07	-.015 (.002) -.14 to .13	.077 (-.002) -.14 to .28	.117 (-.001) -.08 to .31	-.021 (.000) -.21 to .17
State Anxiety		.113 (-.002) -.07 to .29	.106 (.001) -.08 to .29	.076 (-.004) -.11 to .24	-.145 (.001) -.32 to .03	.065 (-.001) -.13 to .26	.208+ (.000) .02 to .38	.052 (.002) -.13 to .24	-.007 (.002) -.20 to .20	.101 (.002) -.08 to .29	.084 (.001) -.10 to .26	.070 (.000) -.11 to .25
Trait Anxiety	.113 (-.002) -.07 to .29		.779* (.000) .72 to .84	.448* (-.003) .27 to .60	-.053 (.003) -.24 to .14	.100 (.000) -.08 to .28	.537* (-.002) .40 to .66	-.527* (.000) -.66 to -.38	-.179 (.001) -.37 to .02	.440* (-.002) .26 to .60	.295* (-.001) .09 to .48	-.130 (-.004) -.32 to .06
Depression	.106 (.001) -.08 to .29	.779* (.000) .72 to .84		.345* (-.001) .16 to .51	-.057 (.002) -.24 to .14	.165 (-.003) -.02 to .33	.646* (-.002) .53 to .74	-.523* (.000) -.66 to -.38	-.154 (.000) -.35 to .06	.421* (-.002) .24 to .59	.242+ (-.001) .05 to .43	-.221+ (-.004) -.42 to -.01
Neuroticism	.076 (-.004) -.11 to .24	.448* (-.003) .27 to .60	.345* (-.001) .16 to .51		-.083 (.000) -.26 to .10	.187+ (.001) .01 to .35	.237+ (-.001) .05 to .41	-.359* (.001) -.53 to -.16	-.157 (-.001) -.34 to .03	.281* (-.003) .08 to .45	.189+ (-.002) .004 to .36	-.034 (-.003) -.20 to .12
Extraversion	-.145 (.001) -.32 to .03	-.053 (.003) -.24 to .14	-.057 (.002) -.24 to .14	-.083 (.000) -.26 to .10		-.029 (.001) -.24 to .17	-.063 (.002) -.26 to .14	.132 (-.001) -.05 to .30	.203+ (.003) -.003 to .42	-.107 (-.001) -.28 to .08	-.145 (.000) -.35 to .07	-.185 (.001) -.38 to .03
Psychoticism	.065 (-.001) -.13 to .26	100 (.000) -.08 to .28	165 (-.003) -.02 to .33	.187+ (.001) .01 to .35	-.029 (.001) -.24 to .17		.012 (-.003) -.18 to .20	-.091 (-.002) -.30 to .13	-.036 (.001) -.24 to .17	.077 (-.001) -.11 to .26	.063 (-.003) -.14 to .25	.182 (-.005) -.03 to .39
Catastrophizing	.208+ (.000) .02 to .38	.537* (-.002) .40 to .66	.646* (-.002) .53 to .74	.237+ (-.001) .05 to .41	-.063 (.002) -.26 to .14	.012 (-.003) -.18 to .20		-.265* (.000) -.48 to -.04	-.130 (.001) -.34 to .09	.400* (-.002) .22 to .56	.332* (-.001) .16 to .49	-.018 (-.002) -.19 to.16
Cognitive distraction	.052 (.002) -.13 to .24	-.527* (.000) -.66 to -.38	-.523* (.000) -.66 to -.38	-.359* (.001) -.53 to -.16	.132 (-.001) -.05 to .30	-.091 (-.002) -.30 to .13	-.265* (.000) -.48 to -.04		.503* (-.002) .33 to .64	-.238+ (.000) -.43 to -.03	-.258* (.002) -.46 to -.04	-.040 (.004) -.24 to .17
Ignoring pain sensations	-.007 (.002) -.20 to .20	-.179 (.001) -.37 to .02	-.154 (.000) -.35 to .06	-.157 (-.001) -.34 to .03	.203+ (.003) -.003 to .42	-.036 (.001) -.24 to .17	-.130 (.001) -.34 to .09	.503* (-.002) .33 to .64		-.092 (.001) -.29 to .12	-.178 (.003) -.38 to .04	-.036 (.003) -.26 to .20
Difficulty Identifying Feelings	.101 (.002) -.08 to .29	.440* (-.002) .26 to .60	.421* (-.002) .24 to .59	.281* (-.003) .08 to .45	-.107 (-.001) -.28 to .08	.077 (-.001) -.11 to .26	.400* (-.002) .22 to .56	-.238+ (.000) -.43 to -.03	-.092 (.001) -.29 to .12		.744* (.001) .64 to .83	.172 (-.002) -.05 to .36
Difficulty Describing Feelings	.084 (.001) -.10 to .26	.295* (-.001) .09 to .48	.242+ (-.001) .05 to .43	.189+ (-.002) .004 to .36	-.145 (.000) -.35 to .07	.063 (-.003) -.14 to .25	.332* (-.001) .16 to .49	-.258* (.002) -.46 to -.04	-.178 (.003) -.38 to .04	.744* (.001) .64 to .83		.253* (-.003) .01 to .46
Externally Oriented Thinking	.070 (.000) -.11 to .25	-.130 (-.004) -.32 to .06	-.221+ (-.004) -.42 to -.01	-.034 (-.003) -.20 to .12	-.185 (.001) -.38 to .03	.182 (-.005) -.03 to .39	-.018 (-.002) -.19 to.16	-.040 (.004) -.24 to .17	-.036 (.003) -.26 to .20	.172 (-.002) -.05 to .36	.253* (-.003) .01 to .46	
Fatigue	.151 (-.001) -.03 to .33	.317* (.000) .14 to .48	.318* (.000) .14 to .48	.183 (-.006) -.06 to .40	-.089 (.000) -.26 to .09	.158 (-.002) -.03 to .34	.244+ (.001) .08 to .41	-.196+ (-.003) -.37 to -.02	-.201+ (.001) -.37 to -.02	.274* (.000) .10 to .43	.137 (.001) -.04 to .31	.088 (-.003) -.10 to .26
Fibromyalgia Impact	.156 (.000) -.04 to .35	.449* (-.001) .30 to .59	.531* (-.002) .39 to .65	.168 (-.001) -.02 to .35	-.002 (-.001) -.17 to .16	.118 (-.003) -.07 to .30	.469* (-.001) .33 to .59	-.262* (-.002) -.43 to -.09	-.002 (.001) -.17 to .17	.369* (-.002) .19 to.53	.252* (.001) .08 to .42	.007 (-.002) -.19 to .20
Body Pain	.157 (.003) -.01 to .34	-.419* (-.004) -.55 to -.29	-.488* (-.002) -.62 to -.34	.013 (-.003) -.17 to .18	-.105 (-.005) -.29 to .07	.053 (.001) -.12 to .23	-.224+ (-.001) -.38 to -.06	.241+ (.003) .08 to .40	-.021 (-.002) -.18 to .13	-.223+ (.001) -.39 to -.05	-.086 (-.001) -.25 to .07	.190+ (.004) .03 to .37
Sleep satisfaction	-.144 (.001) -.32 to .04	-.394* (-.003) -.57 to -.22	-.440* (-.004) -.60 to -.28	-.187 (.000) -.37 to .01	.084 (-.004) -.11 to .26	.035 (-.002) -.19 to .26	-.276* (-.004) -.49 to -.06	.266* (-.002) .04 to .47	.073 (-.001) -.12 to .25	-.176 (-.002) -.37 to .02	-.121 (.001) -.32 to .08	.045 (.006) -.15 to .25
Insomnia	-.027 (.000) -.22 to .17	.443* (.002) .27 to .61	.487* (.001) .34 to .63	.173 (.002) -.001 to .35	.016 (.001) -.19 to .23	.016 (.001) -.19 to .23	.280* (.000) .07 to .47	-.331* (.001) -.51 to -.14	-.017 (.002) -.19 to .17	.195+ (.000) .01 to .38	.115 (.000) -.09 to .32	-.100 (-.003) -.32 to .11
Hypersomnia	.162 (.000) -.02 to .34	-.180 (.000) -.36 to .002	-.137 (.001) -.31 to .04	-.001 (-.001) -.18 to .16	-.092 (-.003) -.28 to .08	.038 (-.001) -.15 to .23	-.165 (-.001) -.33 to .000	.185 (-.002) .02 to .35	-.095 (-.003) -.28 to .08	-.032 (-.001) -.22 to .15	-.139 (.000) -.34 to .06	.028 (.003) -.14 to .21
Painful points	.175 (-.001) .002 to .35	.093 (.003) -.07 to .27	.122 (.005) -.07 to .32	.150 (-.001) -.04 to .33	.087 (.006) -.08 to .28	.216+ (-.006) -.01 to .42	.057 (.002) -.08 to .20	-.150 (-.002) -.33 to .04	-.077 (.005) -.23 to .10	.098 (-.001) -.06 to .24	.091 (-.001) -.07 to .25	.149 (-.010) -.08 to .34
Words number	-.022 (.001) -.21 to .18	.511* (-.001) .38 to .62	.535* (-.001) .40 to .66	.202+ (-.004) .02 to .36	-.013 (.002) -.22 to .19	.216+ (-.002) .02 to .39	.483* (-.002) .32 to .63	-.119 (-.001) -.29 to .06	.025 (.001) -.15 to .20	.380* (-.001) .22 to .52	.233+ (.000) .05 to .41	-.109 (-.002) -.31 to .09
Sensorial Pain	-.007 (.002) -.21 to .20	.363* (-.001) .21 to .50	.376* (-.001) .21 to .53	.137 (-.003) -.05 to .29	-.049 (.001) -.26 to .16	.261* (-.002) .08 to .42	.337* (-.002) .14 to .51	-.058 (-.002) -.25 to .13	-.051 (.000) -.23 to .13	.299* (-.001) .13 to .45	.197+ (.000) .01 to .38	-.042 (-.002) -.24 to .15
Emotional Pain	.021 (.001) -.18 to .23	.564* (-.001) .42 to .69	.629* (-.001) .51 to .73	.281* (-.003) .12 to .42	-.255* (.002) -.44 to -.05	.135 (.000) -.08 to .33	.573* (-.002) .44 to .70	-.293* (.001) -.45 to -.13	-.158 (.001) -.34 to .03	.348* (-.001) .16 to .52	.273* (.000) .08 to .45	-.039 (-.003) -.25 to .17
Valorative Pain	.094 (.007) -.15 to .35	-.222+ (-.006) -.42 to -.04	-.214+ (-.004) -.38 to -.07	-.051 (-.004) -.21 to .10	-.067 (-.007) -.27 to .10	.157 (.002) .01 to .31	-.181 (-.002) -.34 to -.03	.064 (.003) -.10 to .25	.062 (-.005) -.17 to .25	-.026 (-.001) -.22 to .17	.084 (-.006) -.12 to .26	.223+ (.003) .05 to .39
Miscellany Pain	-.021 (.000) -.22 to .18	.377* (-.002) .23 to .51	.370* (-.002) .21 to .52	.137 (-.003) -.03 to .29	.007 (.000) -.22 to .22	.293* (-.001) .10 to .47	.302* (-.003) .13 to .47	-.054 (-.002) -.22 to .12	.065 (.001) -.12 to .24	.319* (-.002) .15 to .47	.208+ (-.001) .01 to .39	-.032 (-.001) -.24 to .16
Total Pain	.006 (.000) -.20 to .21	.397* (.000) .24 to .54	.424* (-.001) .27 to .57	.148 (-.003) -.04 to .31	-.099 (.002) -.31 to .11	.268* (-.001) .09 to .42	.371* (-.002) .20 to .53	-.088 (-.002) -.27 to .09	-.049 (.001) -.22 to .12	.334* (-.001) .17 to .48	.207+ (.001) .004 to .40	-.040 (-.002) -.24 to .15
Pain Intensity (MPQ)	.241+ (-.003) .08 to .39	.385* (.002) .22 to .54	.395* (.003) .23 to .56	.141 (.001) -.04 to .32	-.077 (.001) -.27 to .13	.189+ (-.004) -.004 to .36	.321* (.000) .13 to .50	-.156 (-.001) -.34 to .03	-.235+ (.002) -.41 to -.04	.100 (.001) -.09 to .28	.007 (-.001) -.19 to .21	-.073 (-.004) -.28 to .12
Pain Intensity (VAS)	-.091 (.000) -.26 to .09	.585* (-.001) .47 to .69	.643* (-.001) .53 to .74	.139 (-.001) -.02 to .29	.100 (.001) -.12 to .31	.114 (-.007) -.11 to .31	.478* (-.001) .34 to .61	-.249* (.001) -.40 to -.09	-.104 (.004) -.30 to .11	.204+ (-.002) .004 to .39	.115 (-.001) -.10 to .32	-.160 (-.006) -.42 to .10

Note: + $p < 0.05$, * $p < 0.01$, two-tailed test.

Table 3S. Correlations (Pearson Coefficient (bootstrap bias) bootstrap 95% CIs) in Healthy Participants.

	Antidepressant	Anxiolytic	Non opioid analgesic	Opiate analgesic	Analgesic	Fatigue	Fibromyalgia Impact	Body Pain	Sleep satisfaction	Insomnia	Hypersomnia	Painful points	Words number	Sensorial Pain	Emotional Pain	Valorative Pain	Miscellany Pain	Total Pain	Pain Intensity (MPQ)	Pain Intensity (VAS)
Antidepressant		1.00* (.000) 1.00 to 1.00				.303 (.069) .21 to .61	-.092 (-.023) -.20 to -.06	-.370 (-.128) -.97 to -.22	.149 (.035) .10 to .32	.044 (.040) -.04 to .43	.149 (.035) .10 to .32	.058 (.048) -.02 to .41	.544* (.077) .46 to .82	.471+ (.083) .37 to .79	.517* (.097) .36 to .89	.082 (.018) .04 to .18	.685* (.070) .57 to .93	.574* (.077) .48 to .85	.351 (.083) .26 to .69	.011 (.011) -.08 to .17
Anxiolytic	1.00* (.000) 1.00 to 1.00					.303 (.069) .21 to .61	-.092 (-.023) -.20 to -.06	-.370 (-.128) -.97 to -.22	.149 (.035) .10 to .32	.044 (.040) -.04 to .43	.149 (.035) .10 to .32	.058 (.048) -.02 to .41	.544* (.077) .46 to .82	.471+ (.083) .37 to .79	.517* (.097) .36 to .89	.082 (.018) .04 to .18	.685* (.070) .57 to .93	.574* (.077) .48 to .85	.351 (.083) .26 to .69	.011 (.011) -.08 to .17
Non opioid analgesic																				
Opiate analgesic																				
Analgesic																				
State Anxiety	-.060 (-.010) -.16 to .02	-.060 (-.010) -.16 to .02				-.107 (.041) -.34 to .39	.012 (.038) -.15 to .40	.216 (-.002) -.06 to .45	.106 (-.045) -.47 to .33	.006 (.047) -.14 to .42	.106 (-.045) -.47 to .33	-.179 (.005) -.37 to .15	-.122 (.039) -.34 to .35	-.131 (.038) -.36 to .37	-.085 (.002) -.23 to .08	.039 (-.032) -.32 to .17	-.062 (.049) -.26 to .46	-.115 (.037) -.33 to .36	.033 (.025) -.13 to .42	-.165 (-.002) -.33 to .02
Trait Anxiety	.247 (.058) .17 to .52	.247 (.058) .17 to .52				.228 (.005) -.14 to .59	-.104 (.003) -.35 to .17	.144 (-.038) -.47 to .45	-.296 (.002) -.60 to .04	.406+ (-.003).09 to .69	-.296 (.002) -.60 to .04	.239 (-.003) -.07 to .49	.639* (.002) .44 to .80	.615* (.004) .40 to .79	.483+ (-.021) .08 to .74	.098 (-.005) -.18 to .39	.571* (.014) .42 to .75	.635* (.006) .44 to .80	.496* (-.007) .16 to .74	.118 (.004) -.19 to .42
Depression	.409+ (.126) .24 to .88	.409+ (.126) .24 to .88				.158 (.039) -.15 to .61	-.122 (.007) -.29 to .12	-.049 (-.034) -.69 to .24	-.231 (.037) -.57 to .25	547* (-.093) -.14 to .91	-.231 (.037) -.57 to .25	.014 (.020) -.18 to .35	.424+ (-.028) -.17 to .72	.304 (.001) -.10 to .68	.667* (-.131) -.39 to .88	.105 (-.009) -.14 to .27	.399+ (-.005) -.16 to .25	.404+ (-.017) -.14 to .74	.296 (.042) .04 to .70	-.210 (.016) -.44 to .15
Neuroticism	.254 (.058) .19 to .51	.254 (.058) .19 to .51				.213 (.000) -.17 to .56	-.073 (-.012) -.37 to .18	.106 (-.017) -.35 to .39	-.096 (.008) -.45 to .28	.362 (-.011) .03 to .65	-.096 (.008) -.45 to .28	.401+ (.005) .19 to .61	.371 (-.002) -.02 to .68	.373 (.002) .02 to .67	.401+ (-.028) -.14 to .69	-.105 (.010) -.48 to .26	.356 (-.003) .05 to .61	.398+ (.000) .05 to .68	.134 (.013) -.28 to .53	-.150 (.018) -.47 to .27
Extraversion	.067 (.014) -.01 to .18	.067 (.014) -.01 to .18				-.307 (.018) -.66 to .19	-.408+ (.015) -.76 to .18	-.235 (-.004) -.41 to -.08	.209 (-.025) -.36 to .60	-.308 (-.003) -.78 to .24	.209 (-.025) -.36 to .60	.004 (-.044) -.47 to .26	.177 (-.002) -.19 to .48	.184 (-.003) -.13 to .44	.309 (.003) -.03 to .57	.447+ (-.018) -.16 to .81	-.014 (.000) -.43 to .30	.172 (.000) -.18 to .47	.077 (.000) -.28 to .37	-.010 (-.012) -.45 to .34
Psychoticism	.060 (.016) -.02 to .20	.060 (.016) -.02 to .20				.285 (.005) -.03 to .63	.240 (.010) .03 to .48	-.150 (.017) -.41 to .22	-.002 (.002) -.35 to .33	.182 (-.006) -.08 to .43	-.002 (.002) -.35 to .33	.270 (-.005) -.11 to .63	-.053 (-.010) -.47 to .30	.076 (-.016) -.35 to .42	-.128 (-.022) -.56 to .12	-.200 (-.001) -.48 to .08	-.024 (.000) -.45 to .34	.021 (-.013) -.41 to .37	-.034 (.004) -.25 to .23	-.416+ (.016) -.67 to -.04
Catastrophizing	.835* (.047) .69 to 1.00	.835* (.047) .69 to 1.00				.175 (-.048) -.26 to .53	-.126 (.001) -.24 to -.01	-.255 (.030) -.88 to .18	.151 (-.024) -.25 to .34	-.031 (-.004) -.22 to .29	.151 (-.024) -.25 to .34	-.027 (-.005) -.24 to .28	.441+ (-.080) -.23 to .79	.363 (-.063) -.24 to .74	.474+ (-.038) -.06 to .87	.134 (.003) .06 to .24	.512* (-.150) -.26 to .87	.450+ (-.095) -.23 to .81	.270 (-.044) -.15 to .63	.113 (.037) -.14 to .66
Cognitive distraction	-.148 (-.034) -.31 to -.11	-.148 (-.034) -.31 to -.11				-.287 (.007) -.55 to .002	-.246 (.014) -.44 to .05	.255 (-.013) -.02 to .43	-.088 (-.005) -.49 to .29	.135 (-.012) -.27 to .57	-.088 (-.005) -.49 to .29	-.263 (.003) -.44 to -.02	.144 (.016) -.20 to .56	.018 (.016) -.28 to .40	.244 (-.011) -.21 to .62	.170 (-.007) -.13 to .39	.108 (.036) -.20 to .57	.079 (.021) -.23 to .50	.074 (.001) -.23 to .36	.162 (-.013) -.31 to .60
Ignoring pain sensations	-.144 (-.032) -.31 to -.10	-.144 (-.032) -.31 to -.10				-.266 (.007) -.54 to .06	-.261 (.011) -.45 to -.01	.232 (-.019) -.09 to .42	.354 (-.007) .07 to .60	-.234 (.013) -.41 to .01	.354 (-.007) .07 to .60	.026 (.026) -.29 to .51	.069 (.014) -.26 to .49	.092 (.014) -.26 to .52	-.018 (.031) -.29 to .49	.280 (-.006) .12 to .46	.114 (.026) -.23 to .58	.098 (.018) -.25 to .55	.124 (-.002) -.22 to .45	.125 (.006) -.24 to .50
Difficulty Identifying Feelings	.322 (.073) .23 to .63	.322 (.073) .23 to .63				-.038 (.005) -.40 to .38	-.328 (.006) -.52 to -.07	.043 (-.031) -.49 to .33	.004 (-.009) -.41 to .36	.206 (.006) -.21 to .63	.004 (-.009) -.41 to .36	.327 (.017) .02 to .64	.685* (-.005) .46 to .83	.667* (.001) .46 to .83	.610* (-.007) .27 to .81	.233 (-.004) -.06 to .45	.725* (.003) .54 to .86	.732* (.000) .55 to .86	.402+ (.000) .05 to .70	.046 (.012) -.30 to .49
Difficulty Describing Feelings	.210 (.056) .13 to .50	.210 (.056) .13 to .50				-.052 (.006) -.41 to .35	-.331 (.000) -.52 to -.15	.185 (-.018) -.25 to .43	-.164 (-.006) -.59 to .24	.224 (.015) -.21 to .63	-.164 (-.006) -.59 to .24	.486+ (-.027) -.10 to .75	.479+ (-.002) .15 to .71	.540* (-.005) .21 to .77	.455+ (-.002) .07 to .72	.041 (.002) -.38 to .35	.432+ (.005) .15 to .66	.541* (.000) .24 to .75	.140 (.004) -.17 to .44	-.144 (.017) -.43 to .27
Externally Oriented Thinking	.286 (.062) .23 to .54	.286 (.062) .23 to .54				.033 (.006) -.38 to .49	-.379 (.006) -.61 to -.07	.150 (-.039) -.47 to .48	.024 (-.008) -.37 to .39	.078 (.025) -.30 to .48	.024 (-.008) -.37 to .39	.347 (-.007) -.08 to .60	757* (.002) .61 to .87	.762* (.000) .61 to .89	.474+ (.005) .23 to .70	.282 (-.005) -.06 to .55	.667* (.015) 54 to .82	.763* (.004) .63 to .88	.461+ (-.007) .10 to .71	.084 (.002) -.21 to .36
Fatigue	.303 (.069) .21 to .61	.303 (.069) .21 to .61					.673* (-.015) .33 to .88	.146 (-.027) -.40 to .44	-.405+ (.006) -.75 to .002	.240 (.004) -.16 to .66	-.405+ (.006) -.75 to .002	.363 (-.001) -.09 to .67	.162 (-.009) -.26 to .55	.221 (-.002) -.19 to .61	-.022 (-.015) -.44 to .39	-.331 (.001) -.67 to .03	.184 (-.020) -.23 to .57	.186 (-.010) -.24 to .58	.218 (.001) -.24 to .66	-.092 (.000) -.40 to .29
Fibromyalgia Impact	-.092 (-.023) -.20 to -.06	-.092 (-.023) -.20 to -.06				.673* (-.015) .33 to .88		.175 (-.005) .01 to .30	-.502* (.035) -.78 to .16	.319 (-.005) -.16 to .78	-.502* (.035) -.78 to .16	.051 (-.002) -.29 to .59	-.236 (-.007) -.51 to .04	-.232 (-.003) -.45 to -.01	-.283 (-.019) -.49 to -.13	-.383+ (-.014) -.89 to .11	-.154 (.002) -.34 to .08	-.245 (-.005) -.47 to -.02	.066 (-.021) -.56 to .60	.041 (-.021) -.43 to .51
Body Pain	-.370 (-.128) -.97 to -.22	-.370 (-.128) -.97 to -.22				.146 (-.027) -.40 to .44	.175 (-.005) .01 to .30		-.294 (.003) -.47 to -.12	.158 (-.008) -.09 to .31	-.294 (.003) -.47 to -.12	.088 (-.014) -.18 to .23	-.056 (-.014) -.68 to .34	-.024 (-.026) -.64 to .33	-.138 (-.008) -.66 to .17	-.179 (-.001) -.30 to -.08	-.170 (-.007) -.84 to .26	-.086 (-.015) -.72 to .31	-.183 (-.038) -.65 to .06	-.037 (-.020) -.31 to .12
Sleep satisfaction	.149 (.035) .10 to .32	.149 (.035) .10 to .32				-.405+ (.006) -.75 to .002	-.502* (.035) -.78 to .16	-.294 (.003) -.47 to -.12		-.620* (.012) -.81 to -.27	1.00* (.000) 1.00 to 1.00	-.141 (.012) -.48 to .30	-.031 (-.015) -.48 to .34	-.039 (-.009) -.43 to .31	-.086 (.023) -.53 to .39	.455+ (-.009) -.12 to .79	.079 (-.023) -.34 to .35	-.003 (-.016) -.43 to .34	-.014 (-.005) -.46 to .41	.003 (.004) -.41 to .43
Insomnia	.044 (.040) -.04 to .43	.044 (.040) -.04 to .43				.240 (.004) -.16 to .66	.319 (-.005) -.16 to .78	.158 (-.008) -.09 to .31	-.620* (.012) -.81 to -.27		-.620* (.012) -.81 to -.27	.043 (.033) -.10 to .37	.119 (.018) -.30 to .54	.033 (.026) -.25 to .47	.365 (-.076) -.35 to .82	-.342 (.000) -.88 to .19	.103 (.032) -.14 to .51	.090 (.021) -.26 to .50	.094 (.034) -.11 to .58	-.128 (.028) -.37 to .30
Hypersomnia	.149 (.035) .10 to .32	.149 (.035) .10 to .32				-.405+ (.006) -.75 to .002	-.502* (.035) -.78 to .16	-.294 (.003) -.47 to -.12	1.00* (.000) 1.00 to 1.00	-.620* (.012) -.81 to -.27		-.141 (.012) -.48 to .30	-.031 (-.015) -.48 to .34	-.039 (-.009) -.43 to .31	-.086 (.023) -.53 to .39	.455+ (-.009) -.12 to .79	.079 (-.023) -.34 to .35	-.003 (-.016) -.43 to .34	-.014 (-.005) -.46 to .41	.003 (.004) -.41 to .43
Painful points	.058 (.048) -.02 to .41	.058 (.048) -.02 to .41				.363 (-.001) -.09 to .67	.051 (-.002) -.29 to .59	.088 (-.014) -.18 to .23	-.141 (.012) -.48 to .30	.043 (.033) -.10 to .37	-.141 (.012) -.48 to .30		.393+ (.018) .12 to .69	.489* (.006) .05 to .81	.293 (.032) .13 to .64	.165 (.003) -.02 to .34	.143 (.050) -.10 to .57	.408+ (.024) .10 to .74	.112 (.033) -.18 to .61	.018 (.034) -.19 to .46
Words number	.544* (.077) .46 to .82	.544* (.077) .46 to .82				.162 (-.009) -.26 to .55	-.236 (-.007) -.51 to .04	-.056 (-.014) -.68 to .34	-.031 (-.015) -.48 to .34	.119 (.018) -.30 to .54	.031 (-.015) -.48 to .34	.393+ (.018) .12 to .69		.951* (.001) .91 to .98	.752* (.002) .54 to .92	.424+ (.000) .22 to .62	.857* (-.011) .67 to .96	.982* (-.001) .96 to .99	.583* (-.012) .28 to .76	.316 (.004) .05 to .60
Sensorial Pain	.471+ (.083) .37 to .79	.471+ (.083) .37 to .79				.221 (-.002) -.19 to .61	-.232 (-.003) -.45 to -.01	-.024 (-.026) -.64 to .33	-.039 (-.009) -.43 to .31	.033 (.026) -.25 to .47	-.039 (-.009) -.43 to .31	.489* (.006) .05 to .81	.951* (.001) .91 to .98		.595* (.016) .33 to .87	.351 (-.001) .17 to .53	.798* (-.002) .57 to .94	976* (.002) .96 to .100	.580* (-.010) .26 to .79	.249 (.009) .01 to .54
Emotional Pain	.517* (.097) .36 to .89	.517* (.097) .36 to .89				-.022 (-.015) -.44 to .39	-.283 (-.019) -.49 to -.13	-.138 (-.008) -.66 to .17	-.086 (.023) -.53 to .39	.365 (-.076) -.35 to .82	-.086 (.023) -.53 to .39	.293 (.032) .13 to .64	.752* (.002) .54 to .92	.595* (.016) .33 to .87		.348 (.013) .19 to .56	.574* (-.028) .13 to .86	.698* (.004) .45 to .91	.244 (-.004) -.12 to .59	.219 (.031) -.16 to .76
Valorative Pain	.082 (.018) .04 to .18	.082 (.018) .04 to .18				-.331 (.001) -.67 to .03	-.383+ (-.014) -.89 to .11	-.179 (-.001) -.30 to -.08	.455+ (-.009) -.12 to .79	-.342 (.000) -.88 to .19	.455+ (-.009) -.12 to .79	.165 (.003)-.02 to .34	.424+ (.000) .22 to .62	.351 (-.001) .17 to .53	.348 (.013) .19 to .56		.234 (.003) .10 to .40	.372 (.002) .19 to .56	.513* (-.017) .10 to .76	.291 (-.005) -.11 to .58
Miscellany Pain	.685* (.070) .57 to .93	.685* (.070) .57 to .93				.184 (-.020) -.23 to .57	-.154 (.002) -.34 to .08	-.170 (-.007) -.84 to .26	.079 (-.023) -.34 to .35	.103 (.032) -.14 to .51	.079 (-.023) -.34 to .35	.143 (.050) -.10 to .57	.857* (-.011) .67 to .96	.798* (-.002) .57 to .94	.574* (-.028) .13 to .86	.234 (.003) .10 to .40		.892* (-.010) .72 to .97	.576* (.002) .24 to .80	.122 (.016) -.12 to .45
Total Pain	.574* (.077) .48 to .85	.574* (.077) .48 to .85				.186 (-.010) -.24 to .58	-.245 (-.005) -.47 to -.02	-.086 (-.015) -.72 to .31	-.003 (-.016) -.43 to .34	.090 (.021) -.26 to .50	-.003 (-.016) -.43 to .34	.408+ (.024) .10 to .74	.982* (-.001) .96 to .99	.976* (.002) .96 to .100	.698* (.004) .45 to .91	.372 (.002) .19 to .56	.892* (-.010) .72 to .97		.592* (-.011) .27 to .78	.236 (.011) .001 to .51
Pain Intensity (MPQ)	.351 (.083) .26 to .69	.351 (.083) .26 to .69				.218 (.001) -.24 to .66	.066 (-.021) -.56 to .60	-.183 (-.038) -.65 to .06	-.014 (-.005) -.46 to .41	.094 (.034) -.11 to .58	-.014 (-.005) -.46 to .41	.112 (.033) -.18 to .61	.583* (-.012) .28 to .76	.580* (-.010) .26 to .79	.244 (-.004) -.12 to .59	.513* (-.017) .10 to .76	.576* (.002) .24 to .80	.592* (-.011) .27 to .78		.387+ (.010) .03 to .76
Pain Intensity (VAS)	.011 (.011) -.08 to .17	.011 (.011) -.08 to .17				-.092 (.000) -.40 to .29	.041 (-.021) -.43 to .51	-.037 (-.020) -.31 to .12	.003 (.004) -.41 to .43	-.128 (.028) -.37 to .30	.003 (.004) -.41 to .43	.018 (.034) -.19 to .46	.316 (.004) .05 to .60	.249 (.009) .01 to .54	.219 (.031) -.16 to .76	.291 (-.005) -.11 to .58	.122 (.016) -.12 to .45	.236 (.011) .001 to .51	.387+ (.010) .03 to .76	

Note: + $p < 0.05$, * $p < 0.01$, two-tailed test.

Table 4S. Correlations (Pearson Coefficient (bootstrap bias) bootstrap 95% CIs) in Healthy Participants.

Variables	State Anxiety	Trait Anxiety	Depression	Neuroticism	Extraversion	Psychoticism	Catastrophizing	Cognitive distraction	Ignoring pain sensations	Difficulty Identifying Feelings	Difficulty Describing Feelings	Externally Oriented Thinking
Antidepressant	-.060 (-.010) -.16 to .02	.247 (.058) .17 to .52	.409+ (.126) .24 to .88	.254 (.058) .19 to .51	.067 (.014) -.01 to .18	.060 (.016) -.02 to .20	.835* (.047) .69 to 1.00	-.148 (-.034) -.31 to -.11	-.144 (-.032) -.31 to -.10	.322 (.073) .23 to .63	.210 (.056) .13 to .50	.286 (.062) .23 to .54
Anxiolytic	-.060 (-.010) -.16 to .02	.247 (.058) .17 to .52	.409+ (.126) .24 to .88	.254 (.058) .19 to .51	.067 (.014) -.01 to .18	.060 (.016) -.02 to .20	.835* (.047) .69 to 1.00	-.148 (-.034) -.31 to -.11	-.144 (-.032) -.31 to -.10	.322 (.073) .23 to .63	.210 (.056) .13 to .50	.286 (.062) .23 to .54
Non opioid analgesic												
Opiate analgesic												
Analgesic												
State Anxiety		.023 (.054) -.25 to .61	.171 (.053) .02 to .64	-.297 (.050) -.56 to .28	.241(-.019) -.14 to .43	.101 (-.006) -.21 to .38	.230 (.010) -.18 to .88	.579* (-.032) .04 to .86	.484+ (-.019) .08 to .74	-.001 (.024) -.18 to .30	.102 (-.008) -.18 to .31	.295 (.025) .12 to .59
Trait Anxiety	.023 (.054) -.25 to .61		.590* (-.007) .25 to .76	.435+ (.000) .02 to .78	.205 (.004) -.14 to .54	-.092 (-.004) -.40 to .22	.139 (-.041) -.25 to .45	.302 (.008) -.04 to .65	.221 (-.004) -.16 to .56	.656* (-.001) .38 to .85	.430+ (.005) .09 to .74	.693* (-.001) .46 to .86
Depression	.171 (.053) .02 to .64	.590* (-.007) .25 to .76		.361 (-.052) -.26 to .65	.269 (.002) .01 to .47	.097 (-.001) -.18 to .34	.332 (-.032) -.33 to .84	.297 (-.010) -.20 to .69	-.067 (.033) -.33 to .43	.492* (-.006) -.04 to .83	.407+ (-.001) .03 to .72.	.428+ (.019) .20 to .69
Neuroticism	-.297 (.050) -.56 to .28	.435+ (.000) .02 to .78	.361 (-.052) -.26 to .65		-.027 (-.003) -.36 to .30	.310 (-.008) -.05 to .60	.081 (-.049) -.42 to .43	-.111 (.006) -.49 to .32	.023 (.011) -.32 to .41	.475+ (.001) .16 to .74	.405+ (-.002) .08 to .67	.262 (.005) -.12 to .62
Extraversion	.241 (-.019) -.14 to .43	.205 (.004) -.14 to .54	.269 (.002) .01 to .47	-.027 (-.003) -.36 to .30		-.105 (-.005) -.46 to .28	.187 (.020) .06 to .37	.224 (-.003) -.19 to .56	.209 (-.001) -.18 to .52	.051 (.013) -.38 to .46	-.023 (.010) -.45 to .41	.194 (-.001) -.18 to .53
Psychoticism	.101 (-.006) -.21 to .38	-.092 (-.004) -.40 to .22	.097 (-.001) -.18 to .34	.310 (-.008) -.05 to .60	-.105 (-.005) -.46 to .28		.085 (-.014) -.31 to .33	-.274 (-.008) -.63 to .09	-.062 (-.018) -.54 to .33	-.029 (-.005) -.50 to .42	.206 (-.027) -.30 to .59	.010 (-.015) -.43 to .39
Catastrophizing	.230 (.010) -.18 to .88	.139 (-.041) -.25 to .45	.332 (-.032) -.33 to .84	.081 (-.049) -.42 to .43	.187 (.020) .06 to .37	.085 (-.014) -.31 to .33		.177 (.073) -.21 to .75	.082 (.058) -.22 to .59	.206 (-.055) -.26 to .55	.167 (-.028) -.26 to .47	.291 (-.025) -.02 to .54
Cognitive distraction	.579* (-.032) .04 to .86	.302 (.008) -.04 to .65	.297 (-.010) -.20 to .69	-.111 (.006) -.49 to .32	.224 (-.003) -.19 to .56	-.274 (-.008) -.63 to .09	.177 (.073) -.21 to .75		.557* (-.005) .19 to .84	.230 (.007) -.14 to .62	.168 (.013) -.19 to .57	.383+ (.005) .12 to .66
Ignoring pain sensations	.484+ (-.019) .08 to .74	.221 (-.004) -.16 to .56	-.067 (.033) -.33 to .43	.023 (.011) -.32 to .41	.209 (-.001) -.18 to .52	-.062 (-.018) -.54 to .33	.082 (.058) -.22 to .59	.557* (-.005) .19 to .84		.362 (-.007) -.05 to .71	.226 (-.009) -.18 to .58	.409+ (-.002) .11 to .70
Difficulty Identifying Feelings	-.001 (.024) -.18 to .30	.656* (-.001) .38 to .85	.492* (-.006) -.04 to .83	.475+ (.001) .16 to .74	.051 (.013) -.38 to .46	-.029 (-.005) -.50 to .42	.206 (-.055) -.26 to .55	.230 (.007) -.14 to .62	.362 (-.007) -.05 to .71		.803* (.003) .63 to .93	.729* (.002) .51 to .89
Difficulty Describing Feelings	.102 (-.008) -.18 to .31	.430+ (.005) .09 to .74	.407+ (-.001) .03 to .72	.405+ (-.002) .08 to .67	-.023 (.010) -.45 to .41	.206 (-.027) -.30 to .59	.167 (-.028) -.26 to .47	.168 (.013) -.19 to .57	.226 (-.009) -.18 to .58	.803* (.003) .63 to .93		.611* (-.001) .32 to .85
Externally Oriented Thinking	.295 (.025) .12 to .59	.693* (-.001) .46 to .86	.428+ (.019) .20 to .69	.262 (.005) -.12 to .62	.194 (-.001) -.18 to .53	.010 (-.015) -.43 to .39	.291 (-.025) -.02 to .54	.383+ (.005) .12 to .66	.409+ (-.002) .11 to .70	.729* (.002) .51 to .89	.611* (-.001) .32 to .85	
Fatigue	-.107 (.041) -.34 to .39	.228 (.005) -.14 to .59	.158 (.039) -.15 to .61	.213 (.000) -.17 to .56	-.307 (.018) -.66 to .19	.285 (.005) -.03 to .63	.175 (-.048) -.26 to .53	-.287 (.007) -.55 to .002	-.266 (.007) -.54 to .06	-.038 (.005) -.40 to .38	-.052 (.006) -.41 to .35	.033 (.006) -.38 to .49
Fibromyalgia Impact	.012 (.038) -.15 to .40	-.104 (.003) -.35 to .17	-.122 (.007) -.29 to .12	-.073 (-.012) -.37 to .18	-.408+ (.015) -.76 to .18	.240 (.010) .03 to .48	-.126 (.001) -.24 to -.01	-.246 (.014) -.44 to .05	-.261 (.011) -.45 to -.01	-.328 (.006) -.52 to -.07	-.331 (.000) -.52 to -.15	-.379 (.006) -.61 to -.07
Body Pain	.216 (-.002) -.06 to .45	.144 (-.038) -.47 to .45	-.049 (-.034) -.69 to .24	.106 (-.017) -.35 to .39	-.235 (-.004) -.41 to -.08	-.150 (.017) -.41 to .22	-.255 (.030) -.88 to .18	.255 (-.013) -.02 to .43	.232 (-.019) -.09 to .42	.043 (-.031) -.49 to .33	.185 (-.018) -.25 to .43	.150 (-.039) -.47 to .48
Sleep satisfaction	.106 (-.045) -.47 to .33	-.296 (.002) -.60 to .04	-.231 (.037) -.57 to .25	-.096 (.008) -.45 to .28	.209 (-.025) -.36 to .60	-.002 (.002) -.35 to .33	.151 (-.024) -.25 to .34	-.088 (-.005) -.49 to .29	.354 (-.007) .07 to .60	.004 (-.009) -.41 to .36	-.164 (-.006) -.59 to .24	.024 (-.008) -.37 to .39
Insomnia	.006 (.047) -.14 to .42	.406+ (-.003) .09 to .69	.547* (-.093) -.14 to .91	.362 (-.011) .03 to .65	-.308 (-.003) -.78 to .24	.182 (-.006) -.08 to .43	-.031 (-.004) -.22 to .29	.135 (-.012) -.27 to .57	-.234 (.013) -.41 to .01	.206 (.006) -.21 to .63	.224 (.015) -.21 to .63	.078 (.025) -.30 to .48
Hypersomnia	.106 (-.045) -.47 to .33	-.296 (.002) -.60 to .04	-.231 (.037) -.57 to .25	-.096 (.008) -.45 to .28	.209 (-.025) -.36 to .60	-.002 (.002) -.35 to .33	.151 (-.024) -.25 to .34	-.088 (-.005) -.49 to .29	.354 (-.007) .07 to .60	.004 (-.009) -.41 to .36	-.164 (-.006) -.59 to .24	.024 (-.008) -.37 to .39
Painful points	-.179 (.005) -.37 to .15	.239 (-.003) -.07 to .49	.014 (.020) -.18 to .35	.401+ (.005) .19 to .61	.004 (-.044) -.47 to .26	.270 (-.005) -.11 to .63	-.027 (-.005) -.24 to .28	-.263 (.003) -.44 to -.02	.026 (.026) -.29 to .51	.327 (.017) .02 to .64	.486+ (-.027) -.10 to .75	.347 (-.007) -.08 to .60
Words number	-.122 (.039) -.34 to .35	.639* (.002) .44 to .80	.424+ (-.028) -.17 to .72	.371 (-.002) -.02 to .68	.177 (-.002) -.19 to .48	-.053 (-.010) -.47 to .30	.441+ (-.080) -.23 to .79	.144 (.016) -.20 to .56	.069 (.014) -.26 to .49	.685* (-.005) .46 to .83	.479+ (-.002) .15 to .71	757* (.002) .61 to .87
Sensorial Pain	-.131 (.038) -.36 to .37	.615* (.004) .40 to .79	.304 (.001) -.10 to .68	.373 (.002) .02 to .67	.184 (-.003) -.13 to .44	.076 (-.016) -.35 to .42	.363 (-.063) -.24 to .74	.018 (.016) -.28 to .40	.092 (.014) -.26 to .52	.667* (.001) .46 to .83	.540* (-.005) .21 to .77	.762* (.000) .61 to .89
Emotional Pain	-.085 (.002) -.23 to .08	.483+ (-.021) .08 to .74	.667* (-.131) -.39 to .88	.401+ (-.028) -.14 to .69	.309 (.003) -.03 to .57	-.128 (-.022) -.56 to .12	.474+ (-.038) -.06 to .87	.244 (-.011) -.21 to .62	-.018 (.031) -.29 to .49	.610* (-.007) .27 to .81	.455+ (-.002) .07 to .72	.474+ (.005) .23 to .70
Valorative Pain	.039 (-.032) -.32 to .17	.098 (-.005) -.18 to .39	.105 (-.009) -.14 to .27	-.105 (.010) -.48 to .26	.447+ (-.018) -.16 to .81	-.200 (-.001) -.48 to .08	.134 (.003) .06 to .24	.170 (-.007) -.13 to .39	.280 (-.006) .12 to .46	.233 (-.004) -.06 to .45	.041 (.002) -.38 to .35	.282 (-.005) -.06 to .55
Miscellany Pain	-.062 (.049) -.26 to .46	.571* (.014) .42 to .75	.399+ (-.005) -.16 to .83	.356 (-.003) .05 to .61	-.014 (.000) -.43 to .30	-.024 (.000) -.45 to .34	.512* (-.150) -.26 to .87	.108 (.036) -.20 to .57	.114 (.026) -.23 to .58	.725* (.003) .54 to .86	.432+ (.005) .15 to .66	.667* (.015) 54 to .82
Total Pain	-.115 (.037) -.33 to .36	.635* (.006) .44 to .80	.404+ (-.017) -.14 to .74	.398+ (.000) .05 to .68	.172 (.000) -.18 to .47	.021 (-.013) -.41 to .37	.450+ (-.095) -.23 to .81	.079 (.021) -.23 to .50	.098 (.018) -.25 to .55	.732* (.000) .55 to .86	.541* (.000) .24 to .75	.763* (.004) .63 to .88
Pain Intensity (MPQ)	.033 (.025) -.13 to .42	.496* (-.007) .16 to .74	.296 (.042) .04 to .70	.134 (.013) -.28 to .53	.077 (.000) -.28 to .37	-.034 (.004) -.25 to .23	.270 (-.044) -.15 to .63	.074 (.001) -.23 to .36	.124 (-.002) -.22 to .45	.402+ (.000) .05 to .70	.140 (.004) -.17 to .44	.461+ (-.007) .10 to .71
Pain Intensity (VAS)	-.165 (-.002) -.33 to .02	.118 (.004) -.19 to .42	-.210 (.016) -.44 to .15	-.150 (.018) -.47 to .27	-.010 (-.012) -.45 to .34	-.416+ (.016) -.67 to -.04	.113 (.037) -.14 to .66	.162 (-.013) -.31 to .60	.125 (.006) -.24 to .50	.046 (.012) -.30 to .49	-.144 (.017) -.43 to .27	.084 (.002) -.21 to .36

Note: + $p < 0.05$, * $p < 0.01$, two-tailed test.
